# Supplementary material for: Widespread Shortening of 3’ Untranslated Regions and Increased Exon Inclusion Are Evolutionarily Conserved Features of Innate Immune Responses to Infection
Source: PLoS Genet. 2016 Sep 30;12(9):e1006338. doi: 10.1371/journal.pgen.1006338 (PMC5045211; doi:10.1371/journal.pgen.1006338)
Supplement: S1 Text — (PDF) [file pgen.1006338.s001.pdf]

Supplemental information for: Widespread shortening of 3′ untranslated regions and increased exon inclusion are evolutionarily conserved features of innate immune response to infection

Athma A. Pai*,†, Golshid Baharian*, Ariane Pagé Sabourin, Jessica F. Brinkworth, Yohann Nédélec, Joseph W. Foley, Jean-Christophe Grenier, Katie Siddle, Anne Dumaine, Vania Yotova, Zachary P. Johnson, Robert E. Lanford, Christopher B. Burge, and Luis B. Barreiro†

*These authors contributed equally.

†To whom correspondence should be addressed: athma@mit.edu, luis.barreiro@umontreal.ca

Contents

1 Supplementary Methods **3**

1.1 Experimental Methods 3

1.1.1 Sample collection 3

1.1.2 Bacterial preparation 3

1.1.3 Isolation and infection of macrophages 3

1.1.4 BrdU Cell Proliferation Assay 4

1.2 Whole Blood Sample Collection and Stimulation 4

1.2.1 RNA extraction, library preparation, and sequencing 4

1.2.2 3’RNA library preparation 5

1.2.3 miRNA libraries & sequencing 5
1.2.4 Assessing U1 and totalRNA concentrations 5
1.3 Computational Methods 6
1.3.1 Quantifying gene expression data 6
1.3.2 Differential gene expression following infection 7
1.3.3 Differential isoform usage (DIU) following infection 7
1.3.4 Isoform diversity 11
1.3.5 Analysis of alternative RNA processing events 12
1.3.6 Inter-individual analyses 14
1.3.7 Enrichment of splicing regulatory elements 14
1.3.8 Gene Ontology analyses 15
1.3.9 Analysis of 3'RNA-seq data 15
1.3.10 Analysis of miRNA-seq data 15

2 Supplementary Figures **20**

3 Supplementary Table Legends **36**

1 Supplementary Methods

1.1 Experimental Methods

1.1.1 Sample collection

Buffy coats from 60 healthy donors were obtained from Indiana Blood Center (Indianapolis, IN, USA). Only individuals self-reported as currently healthy, not under medication were included in the study. In addition, each donors blood was tested for standard blood-borne pathogens, and samples were used only if they tested negative for all of pathogens assessed. A signed written consent was obtained from all of the participants and the project was approved by the ethics committee at the CHU Sainte-Justine (protocol #4022). All individuals recruited in this study were males of African-American (N=30) or European-American (N=30) descent between the age of 18 and 55 years old. We decided to focus on only one sex to avoid the potentially confounding effects of potential sex-specific differences in gene expression levels on immune responses to infection.

1.1.2 Bacterial preparation

We infected macrophages with two bacteria, *Salmonella typhimurium* and *Listeria monocytogenes* . The day prior to infection, aliquots of *Salmonella typhimurium* and *Listeria monocytogenes* were thawed and bacteria were grown overnight in Tryptic Soy Broth (TSB) media. Bacterial culture was diluted to mid-log phase prior to infection and supernatant density was checked at OD_{600}.

1.1.3 Isolation and infection of macrophages

Blood mononuclear cells were isolated by Ficoll-Paque centrifugation. Monocytes were purified from peripheral blood mononuclear cells by positive selection with magnetic CD14 MicroBeads (Miltenyi Biotech). Monocytes were then cultured for 7 days in RPMI-1640 (Fisher) supplemented with 10% heat-inactivated FBS (FBS premium, US origin, Wisent), L-glutamine (Fisher) and M-CSF (20ng/mL; R&D systems). Cell cultures were fed every 2 days with complete medium supplemented with the cytokines previously mentioned. The day before the infection, macrophages were harvested to check their differentiation/activation status by flow cytometry, using antibodies against CD1a, CD14, CD83, and HLA-DR (BD Biosciences). Only samples presenting the expected phenotype for non-activated macrophages (CD1a+, CD14+, CD83, and HLA-DRlow) were used in downstream experiments. Macrophages were then re-plated and let to rest for 20-24 hours before the actual infection experiment was performed. Macrophages were infected at a multiplicity of infection (MOI) of 10:1 for *Salmonella typhimurium* and an MOI of 5:1 for *Listeria monocytogenes*. A control group of non-infected macrophages was treated the same way but with only medium without bacteria. After 2 hours in contact with the bacteria, macrophages were washed and cultured for another hour in the presence of 50 μg/ml

gentamycin in order to kill all bacteria that could be present in the medium. The cells were then washed a second time and cultured in complete medium with 3μg/ml gentamycin for an additional 2h and 24h, which are the timepoints to which we refer to in the main text.

1.1.4 BrdU Cell Proliferation Assay

Macrophages and LCL cells were cultured and infected as described above. The amount of 5-bromo-2′-deoxyuridine (BrdU) incorporated into the cells was measured following the manufacturer's recommendations (BrdU Staining Kit for Flow Cytometry FITC, eBioscience). Briefly, 5×10^5 cells were cultured in the presence of BrdU (10 μM) during 2h and 24h after infection. Incorporation of BrdU was measured with an Anti-BrdU FITC Antibody. Flow-cytometric analysis was done with the BD FACSCalibur™.

1.2 Whole Blood Sample Collection and Stimulation

Whole blood was obtained from healthy adult humans (6: 3 males, 3 females, CHU Sainte-Justine), common chimpanzees (6: 3 males, 3 females, Texas Biomedical Research Institute), and rhesus macaques (6: 3 females, 3 males, Yerkes Regional Primate Center). Human samples were acquired with informed consent and with ethics approval from the Research Ethics Board CHU Sainte-Justine (#3557). A signed written consent was acquired from each human participant in the study. Non-human primate samples were humanly acquired in accordance with individual institutional IACUCC requirements. For all species 1 ml of blood was drawn into a 2ml media-containing tube (Truculture tube, Myriad, US) spiked with 3ug of ultrapure LPS from Escherichia coli O111:B (Invivogen, USA) suspended in 60 ul of endotoxin-free water, or 60 ul of endotoxin-free water (negative control). Samples were stimulated with 1ug/ml LPS, at 37C for 4 hours. Total blood leukocytes were isolated via red blood cell lysis buffer and centrifugation at 800x(5 Prime, USA), before being chemically lysed by rigorous vortexing in Qiazol (Qiagen, USA) and frozen at -80C.

1.2.1 RNA extraction, library preparation, and sequencing

Total RNA was extracted from the control and infected/treated samples using the miRNeasy kit (Qiagen), with a DNaseI on-column treatment. RNA quantity was evaluated spectrophotometrically, and the quality was assessed with the Agilent 2100 Bioanalyzer (Agilent Technologies). Only samples with no evidence for RNA degradation (RNA integrity number > 8) were kept for further experiments. RNA-sequencing libraries were prepared using the Illumina TruSeq protocol. Once prepared, indexed cDNA libraries were pooled (6 libraries per pool) in equimolar amounts and the majority were sequenced with single-end 100bp reads on the Illumina HiSeq2500 (9 2hr libraries and all 18 24hr libraries were sequenced with single-end 101 bp reads). This resulted in a mean of 35 million reads per library (Table S1).

1.2.2 3'RNA library preparation

We used Smart-3SEQ (Foley et al, manuscript in preparation), a modified version of the 3SEQ method (*1*). Specifically, the preliminary poly(A) enrichment was omitted and the addition of the second sequencing adapter by dsDNA ligation was replaced with a template-switching cDNA synthesis reaction (*2*).

1.2.3 miRNA libraries & sequencing

miRNA libraries were prepared using the Illumina TruSeq Small RNA library protocol.We multiplexed 36 libraries per lane and sequenced the pool in two lanes with single-end 100bp reads. We obtained an average of 9.6 million raw reads per sample with a minimum yield of 5 million reads.

1.2.4 Assessing U1 and totalRNA concentrations

To investigate the possibility of telescripting, we used qPCR to measure the levels of U1 snRNA in our samples. Telescripting posits that there is a delay in the levels of U1 snRNA relative to immediately increased production of nascent mRNA. We used qRT-PCR procedures and primers as described in (*3*) to quantify levels of U1 snRNA and 5s rRNA (as a control gene, under the assumption that rRNA levels do not change). Changes in 5s rNRA concentrations were calculated by taking the ΔCT value from qPCR measurements across 20 samples. Relative U1 snRNA concentrations were calculated by taking the $\Delta\Delta$CT values from qPCR measurements across 20 samples, where U1 snRNA is measured relative to 5s rRNA concentrations in the same samples.

We were unable to measure nascent RNA levels, but used total RNA concentrations as a proxy. Total RNA concentrations were estimated from Nanodrop measurements of RNA extraction yields across all 60 samples in our study. All samples (both non-infected and infected) were plated at exactly the same macrophage cellular density at the start of the experiment and we have confirmed that these macrophages do not proliferate either before or after infection (Figure S12). While we can therefore rule out increased numbers of cells causing increase total RNA yields in the infected samples, we can not rule out cell death causing fewer numbers of cells from which to extract RNA in the infected samples. Thus, we believe our measurements of increased total RNA concentration are conservative relative to the actual increase in total RNA possibly occuring. While only 1% of total RNA is mRNA, our observations of an approximately 10% increase in total RNA could be caused by a 2-4 fold increase in mRNA concentrations, consistent with (*3*).

1.3 Computational Methods

1.3.1 Quantifying gene expression data

Adaptor sequences and low quality score bases (Phred score < 20) were first trimmed using Trim Galore (version 0.2.7) (for the MISO analyses, this step was skipped since MISO requires all reads to be the same length). The resulting reads were then mapped to the human genome reference sequence (Ensembl GRCh37 release 65) using TopHat (version 2.0.6) and using a hg19 transcript annotation database downloaded from UCSC (date: 2011-08-30) (*4*). We assessed the quality of our RNA-seq libraries and DNA contamination by using RNA-SeQC (*35*) to calculate the rate of intergenic, intragenic, exonic, and intronic coverage in all of our RNA-seq libraries. We see that 98% of mapped reads map to intragenic regions, with only 2% of reads mapping to intergenic regions consistently acorss non-infected, and both sets of infected samples. Furthermore, 93% of reads map to annotated exons. Together, these metrics indicate that there is no systematic bias in genomic DNA contamination between our infection conditions.

Gene-level expression estimates were calculated using featureCounts (version 1.4.6-p3) (*5*). Transcript-level expression values were obtained using RSEM (*6*) and a reference-free aligner, Kallisto (*7*). For the RSEM analysis we started by mapping reads against the genome using STAR (version 2.4.1c) (*21*) in order to obtain aligned reads in transcriptome coordinates. The following parameters were used for index generation (other than default):
–genomeSAindexNbases 2
–genomeChrBinNbits 14
–sjdbOverhang 99

For the alignment we used the following options specifically recommended for downstream analyses with RSEM:
–outSAMattributes NH HI
–outFilterMultimapNmax 20
–outFilterMismatchNmax 999
–outFilterMismatchNoverLmax 0.04
–alignIntronMin 20
–alignIntronMax 1000000
–alignSJoverhangMin 8
–alignSJDBoverhangMin 1
–quantMode TranscriptomeSAM
–runThreadN 12.

Both RSEM and Kallisto were run using default parameters considering the mean (178bp) and standard deviation (58bp) of insert sizes across our RNA-seq libraries.

1.3.2 Differential gene expression following infection

We used DESeq2 (*8*) to identify genes with significant changes in their expression levels upon infection.The analyses were limited to a set of $14,954$ well-annotated Ensembl genes which showed a median gene-expression level of at least 10 reads in at least one of the experimental conditions. For each infection condition, we compared the gene expression levels of the $14,954$ genes between the 60 non-infected and 60 infected samples using the generalized linear model of DESeq2. Specifically, we used the likelihood ratio test in DESeq2 to capture the difference in likelihood between the *full* and the *reduced* model, where the African-American admixture estimate, treatment (infected or non-infected), and batch number for each sample made up the *full* model covariates, while treatment is removed from the *full* model design to yield the *reduced* model. To call a gene significantly differentially expressed, we required a FDR-corrected p-value < 0.001, corresponding to the treatment variable, and an absolute log_2 fold change of mean expression level greater than 0.5. Note that within the above-mentioned differential expression analysis process, and as the libraries may have been sequenced to different depths, raw read counts were normalize by DESeq2-calculated size factors so as to make the samples comparable.

1.3.3 Differential isoform usage (DIU) following infection

To detect genes with differential isoform usage between two groups of non-infected and infected samples, a multivariate generalization of the Welch's t-test is applied (*9*). To this end, we focused on $11,353$ genes out of the above-mentioned $14,954$, which have at least two annotated isoforms and are expressed, in terms of RSEM-estimated gene-level TPM values, in at least half of the samples in each experimental condition. The proportional abundances of each target gene's isoforms are then calculated using RSEM estimations of transcript-level TPM values. Specifically, assuming that the target gene has D isoforms, the relative isoform usage for this gene is denoted by a vector of size D, with its ith element being the proportional abundance of isoform i. Afterwards, an statistical hypothesis test is performed to verify whether the means of two multivariate distributions are equal between the non-infected and infected groups. Suppose that the first group is the group of non-infected samples, and the second group refers to the samples taken from infected cells. Assume that n_i samples have been collected for group i, and let $\pi_{ij} = (\pi_{ij1}, \cdots, \pi_{ijD})$ denote the vector of proportional isoform abundance for sample j of group i, with $i \in \{1, 2\}$ and $j \in \{1, \cdots, n_i\}$. Note that $\sum_{d=1}^{D} \pi_{ijd} = 1$, where π_{ijd} is the relative isoform usage associated with isoform d. The relative isoform usage data is a type of compositional data (*10*), which are vectors representing proportions of a whole that sum to a constant k (in this case, $k = 1$). The space of compositional data with size D is mathematically defined by

$$S^D = \left\{ (x_1, \cdots, x_D) \,\middle|\, x_d > 0 \;\; \forall\, d \in \{1, \cdots, D\}, \; \sum_{d=1}^{D} x_d = k \right\},$$

where S^D is an open simplex (i.e., a generalization of the notion of a two-dimensional triangle to higher dimensions) (*11*). It follows that S^D is not a vector space, since the proportions having a fixed sum leads to dependency between relative isoform usage values within each sample. In fact, the approaches taken in the statistical analysis of compositional data are majorly affected by this characteristic. For one thing, the familiar Euclidean geometry cannot be applied when dealing with compositional data; in particular, although one is used to computing the distance between two real vectors with the standard Euclidean metric, it is not the proper metric to use for compositional data. To illustrate, consider the following pairs of compositions: $\{(0.25, 0.05, 0.7),\ (0.25, 0.1, 0.65)\}$ and $\{(0.25, 0.5, 0.25),\ (0.25, 0.55, 0.2)\}$. The Euclidean distance between the compositions in the first pair equals that of the second pair, as the element-wise difference between the compositions is $(0, 0.05, -0.05)$ for both pairs. However, the second component has doubled in the first pair, while it has only increased by ten percent in the second pair. The fold changes associated with the third components are more comparable between the pairs (0.9 and 0.8 for the first and the second pairs, respectively). In other words, while the Euclidean distances between compositions of both pairs are equal, fold changes imply that the actual distance is larger for the first pair. Therefore, the relative variation of components, rather than their absolute differences, provide the basis to the statistical analysis of compositional data. A commonly-used geometry for compositional data is the Aitchison geometry (*10*), which lends a linear vector space structure to the open simplex and provides us with a way to work with compositional data that is analogous to the real space. Although any multivariate statistical analysis on compositional data can be performed using this vector space structure, it is not easy to follow and sometimes even difficult for the trained eyes. As a result, it is more straightforward to use alternative methods based on transformations of compositional data to the familiar real space. For instance, Aitchison (*12*) proposes a couple of transformations on compositional data, the most important of which are the additive log-ratio transformation (alr) and the centred log-ratio transformation (clr). Neither of these transformations are associated with an orthogonal coordinate system in the simplex; consequently, Egozcue et al. (*11*) introduce the isometric log-ratio transformation (ilr), which maps the log of a given composition to a real vector of size $D-1$, using a set of $D-1$ orthonormal vectors as the basis for S^D. As the distance-preserving ilr transformation removes the inter-dependency between vector elements for each sample, it is yields vectors in $\mathbb{R}^{D-1}$, for which the familiar Euclidean geometry can easily be adopted.

Since there exist more than one orthonormal basis for the simplex, the ilr transformation is not unique. The specific one used in this paper is as follows (*11*). For any $\mathbf{x} = (x_1, \cdots, x_D) \in S^D$,

$$ilr(\mathbf{x}) = log(\mathbf{x}) \times U,$$

where $U = [U_1, \cdots, U_{D-1}]$ is the $D \times (D-1)$ orthonormal basis, with $U_i \in \mathbb{R}^D$ denoting its

ith column:

$$U_{ji} = \begin{cases} \frac{1}{i}\sqrt{\frac{i}{i+1}}, & \text{if } j \leq i \\ -\sqrt{\frac{i}{i+1}}, & \text{if } j = i+1 \\ 0, & \text{otherwise} \end{cases}$$

for $j \in \{1, \cdots, D\}$.

Before applying the ilr transformation on relative isoform usage vectors, the statistical hypothesis test for differential isoform usage between the non-infected and infected groups is initially given by

$$H_0 : \mu_{\pi_1} = \mu_{\pi_2}$$
$$H_1 : \mu_{\pi_1} \neq \mu_{\pi_2},$$

where μ_{π_i} is the mean relative isoform usage for group i. To proceed with the transformation and statistical test, the following two steps are implemented to prepare the data. First, any isoform with an average relative abundance of less than 0.05, across samples, in both groups is removed from statistical testing analysis, to yield biologically meaningful conclusions about DIU. Second, any relative isoform usage value that is remained after this elimination, and is estimated as zero, is then replaced by 0.025 so that all the samples belong to the open simplex S^D. Afterwards, the ilr-transformation is performed on all the samples of both groups, and the differential isoform usage test is modified to

$$H_0 : \mu_{ilr(\pi_1)} = \mu_{ilr(\pi_2)}$$
$$H_1 : \mu_{ilr(\pi_1)} \neq \mu_{ilr(\pi_2)},$$

where $\mu_{ilr(\pi_i)}$ is the mean of ilr-transformed relative isoform usage vectors of group i. Similar to the approach taken by Niu et al. (*13*), we assume that the relative isoform usage data after ilr-transformation has a multivariate normal distribution on $\mathbb{R}^{D-1}$ as follows:

$$ilr(\pi_{i1}), \cdots, ilr(\pi_{in_i}) \sim \mathcal{N}_{D-1}\left(\mu_{ilr(\pi_i)}, \Sigma_i\right) \quad \text{for } i = 1, 2,$$

where Σ_i is the covariance matrix for group i, with $\Sigma_1 \neq \Sigma_2$. Consequently, differential isoform usage boils down to testing the equality of means of two multivariate normal populations, with distinct covariance matrices. This problem is referred to as the multivariate Behrens-Fisher problem, and different approaches have been proposed to tackle the multi-dimensional case. In this paper, we adopted the method proposed by Krishnamoorthy et al. (*14*), which reduces to the well-known Welch's t-test for one-dimensional data (or equivalently, when $D = 2$) (*9*). This test, referred to as T_{KY} herein, cannot be employed when $D - 1 \geq \min\{n_1, n_2\}$ (a case that results in either of the estimated covariance matrices to be singular and non-invertible). This is not a problem in our analysis, since we have a large number of samples per group ($n_1 = n_2 = 60$).

The results of differential isoform usage test is reported in Table S4, where estimation of isoform expression values was done using the RSEM software package. To address DIU effect sizes, Table S4 also includes columns representing the maximum change in relative isoform usage across transcripts of each gene upon either infection. Furthermore, Figure S1C compares these values between the DIU genes at 1% FDR and the background of all tested genes, depicting a significant shift of changes in relative isoform usage towards larger values for DIU genes (Mann-Whitney U test, $P<10^{-15}$ for both *Listeria* and *Salmonella*). To further study the effect of infection on isoform usage, we mathematically define *isoform dominance* by considering the isoforms with the highest and the second highest relative abundance (isoform 1 and isoform 2, respectively) and label isoform 1 dominant if we observe at least a 0.15 difference between the relative abundances of isoform 1 and isoform 2. According to this definition, 75% of DIU genes have a dominant isoform prior to either infection. Following infection with Listeria and Salmonella, 6.2% and 10.2%, respectively, of the aforementioned genes switch to using an alternative dominant isoform. Figure S1E illustrates the percentage of genes with a dominant isoform prior to infection and the proportion of genes switching to an alternative dominant isoform following infection, at different relative abundance thresholds.

We adopted a second method alongside T_{KY} to back-up our numerical results. This method, introduced by Srivastava et al. (*15*) and labeled T_{SKK} here, does not require the estimated covariance matrices to be invertible. In addition, Chen et al. (*16*) propose another method for tackling this problem, relaxing the assumption of multivariate normal distribution for the samples. This method has been proven, theoretically by Srivastava et al. (*15*) and using simulation by Niu et al. (*13*), to be inferior to T_{SKK} in terms of power. As a result, we focus our attention solely on T_{KY} and T_{SKK}. A permutation approach is adopted to control for T_{SKK} type I error, as suggested by Niu et al. (*13*). This issue basically arises from the fact that the null distribution of the test statistic asymptotically converges a standard normal, as $(n_1, n_2, D) \to \infty$ (see (*15*)). For each test (or target gene), we ran 2000 permutations, obtained by shuffling the group labels of samples. For each of the permutations, we computed a new p-value with the new group labels and reported the proportion of p-values less than or equal to the original p-value (with correct group labels) as the modified p-value for that gene. A Spearman correlation of 0.84 and 0.89 was observed between p-values of T_{KY} and p-values of T_{SKK} (with permutations) for infection with Listeria and Salmonella, respectively.

Consistency between DIU results from RSEM and Kallisto isoform estimations. To assess the robustness of our findings of differential isoform usage (DIU), we tested for DIU using isoform-specific expression levels calculated with Kallisto (an alignment-free quantification method (*7*)) at a relaxed FDR threshold of $\leq 5\%$. We observed a 74% and 78% agreement with DIU genes identified using RSEM for *Listeria* and *Salmonella* respectively. This confirms that the identification of DIU genes is largely robust to the method used for isoform quantification.

DIU results considering Transcript Support Level (TSL). We re-conducted the DIU analysis taking into account TSL values, only retaining transcripts passing a conservative TSL filter of

$TSL <= 2$. This obvisouly reduced the number of transcripts considered per gene and also the number of genes tested; however, our new results signified consistency with the trends observed in the initial DIU analysis. Specifically, testing 10222 genes in the new analysis, we observed that among the genes with evidence for significant DIU after infection without considering TSL values, 66% (*Listeria*) and 72% (*Salmonella*) were also identified as DIU (FDR<5%) when constraining on TSL values (Fisher's exact test, $P_{significant\ overlap}<10^{-15}$ for both infections). This verifies that the detection of DIU genes is quite robust to directly adopting a TSL-type metric, given that appropriate filtering steps have been performed beforehand instead. In particular, we limited our original DIU analysis to isoforms with an average relative usage of less than 0.05 TPM across samples, in both groups (non-infected and infected). In fact, this is equivalent to defining an empirical TSL-threshold based on the actual data, thus ensuring that several samples show support for the transcripts in our analysis.

1.3.4 Isoform diversity

Diversity of a data set, which consists of distinct types of individuals, is defined as a function of the number of types present and the evenness (uniformity) with which the individuals are distributed among these types (*17*). Several diversity indices have been proposed to measure the diversity of data sets. In this paper, Shannon entropy H_{sh} (also known as Shannon index (*18*)) was applied to measure the diversity of isoforms for each target gene before and after infection. Specifically, for sample j of group i with D isoforms, Shannon diversity index is calculated by

$$H_{sh} := -\sum_{d=1}^{D} \pi_{ijd} \log(\pi_{ijd}),$$

where H_{sh} quantifies the uncertainty in the prediction of next transcript type, if one is to sequentially observe the isoforms, belonging to the pool of transcripts for a specific gene. If all isoforms are equally common, then $H_{sh} = \log(D)$, while $H_{sh} \to 0$ if all abundance concentrates on only one transcript ($H_{sh} \in (0, \log(D)]$). Therefore, to quantify the change in isoform diversity after infection

$$\Delta_{Shannon} := \frac{H_{sh}^{I} - H_{sh}^{NI}}{\log(D)}$$

was used, with I and NI denoting the infected and non-infected sample, respectively. Positive and negative $\Delta_{Shannon}$ values correspond to genes with an increase or a decrease in their isoform diversity upon infection. We next tested for an association between DE genes and genes showing a large change in isoform diversity following infection. To this end, all genes were divided into 20 bins according to (an increasing order of) their $\Delta_{Shannon}$ values, such that all bins contain the same number of genes. To illustrate, bin one contained genes with the lowest $\Delta_{Shannon}$ values, which were negative, and bin 20 contained genes with the highest $\Delta_{Shannon}$ values, which were positive. The enrichment of down-regulated or up-regulated DE genes among the genes with extreme $\Delta_{Shannon}$ values (i.e., very low and negative, or very high

and positive values) was assessed using logistic regression. For instance, for the high $\Delta_{Shannon}$ case, all genes were classified into two categories based on whether they belonged to bins with very high $\Delta_{Shannon}$ values. Specifically, I_{Δ}^{high} is an indicator function, the value of which equals one if the target gene falls in any of the five bins with the highest $\Delta_{Shannon}$ values (i.e., bins 16, 17, 18, 19, and 20) and zero otherwise. The corresponding logistic regression model was then set up as follows:

$$\log \frac{P\left\{I_{\Delta}^{high} = 1\right\}}{P\left\{I_{\Delta}^{high} = 0\right\}} = \beta_0 + \beta_1 X_1 + \beta_2 X_2,$$

where X_1 (X_2) is an indicator function of whether a gene is both DE and down-regulated (up-regulated). Figure S3B depicts the log_2 of ratio of odds for both low and high $\Delta_{Shannon}$ among the set of up-regulated, down-regulated, and all the DE genes, where the odds ratio for, say, down-regualated DE genes is obtained by

$$\exp(\beta_1) = \left[\frac{P\left\{I_{\Delta}^{high} = 1|X_1 = 1\right\}}{P\left\{I_{\Delta}^{high} = 0|X_1 = 1\right\}}\right]\left[\frac{P\left\{I_{\Delta}^{high} = 0|X_1 = 0\right\}}{P\left\{I_{\Delta}^{high} = 1|X_1 = 0\right\}}\right],$$

with $X_1 = 1$ indicating significant differential expression and down-regulation.
Alongside the Shannon diversity index, the Gini-Simpson diversity index (see (*19*))

$$H_G = 1 - \sum_{d=1}^{D} \pi_{ijd}^2$$

was also adopted to measure the changes in isoform diversity and to verify the results obtained with the Shannon index. A Spearman correlation of 0.94 and 0.95 was observed between $\Delta_{Shannon}$ and Δ_{Gini} values for infection with Listeria and Salmonella, respectively.

1.3.5 Analysis of alternative RNA processing events

Changes across individual RNA processing events were quantified using the MISO software package (v0.4.9) (*20*) using default settings and hg19 version 1 annotations (downloaded from the MISO website, http://miso.readthedocs.org/en/fastmiso/annotation.html). Events were used for further downstream analyses if they were detected in both non-infected and infected conditions in at least 90% of samples. For each sample, a percent spliced in (PSI) value was calculated for each event. Differential RNA processing was estimated using MISO by pairwise comparisons matched non-infected and infected samples to calculate a $\Delta\Psi$ value and a Bayes Factor (BF) for each sample-condition combination. Events were considered to be significantly changing after infection if at least 10% (n $>=$ 6) of samples had a $BF >= 5$ and the $|mean\Delta\Psi| >=$ 0.05 (Table S6).

Analysis of RNA processing after 24hrs. To assess RNA processing changes at a longer time-scale, six out of the 60 initial samples were sequenced after 24hrs of infection with a similar study design (*Listeria*-infected condition, *Salmonella*-infected condition, and a matched non-infected control that was also cultured for 24 hours). To account for differences in power when using only 6 samples, samples sequenced at both 2 hours and 24 hours after infection were analyzed independently of the larger batch of 2 hour samples. Specifically, for each event type, events were used for further downstream analyses if they were detected in both non-infected and infected conditions in 100% of samples at both timepoints. For each sample, a percent spliced in (PSI) value was calculated for each event. Differential RNA processing was estimated using MISO by pairwise comparisons of non-infected and infected samples (using the matched non-infected sample from the corresponding timepoint) to calculate a $\Delta\Psi$ value and a BF for each sample-condition-timepoint combination. Events were considered to be significantly changing after infection if at least 50% (n $\geq$ 3) of samples had a $BF \geq 5$ and the $|mean\Delta\Psi| \geq 0.05$ (Table S6).

Analysis of RNA processing after infection with LPS across primates. RNA-seq reads from human, chimpanzee, and rhesus macaque whole blood cells both before and after infection with LPS were mapped to the genomes of their respective species using Tophat (v2.0.6) as specified above (*4*). For human samples, reads were mapped to the human genome reference sequence (Ensembl GRCh37 release 65, transcript definition version is the same). For chimpanzee samples, reads were mapped to the chimpanzee genome reference sequence (panTro4 - downloaded from UCSC) using a pt4 transcript annotation database from Ensembl and supplemented with annotations derived from Barbosa-Morais *et al.* 2012 (*23*). For rhesus macaque samples, reads were mapped to the rhesus macaque genome reference sequence (rheMac2 - downloaded from UCSC) using a rm2 transcript annotation database from Ensembl and supplemented with annotations derived from Merkin *et al.* 2012 (*24*). Specifically, Tophat (*22*) was used to map each sample, allowing for the discovery of novel junctions and isoforms in addition to junctions already annotated in the provided annotation databases. Cufflinks was then used to identify novel exons, junctions, and isoforms in each sample, and cuffmerge was used to combine across all novel annotation sets. Finally, only isoforms with Cufflinks class codse of ”j” or ”=” were retained for a final annotation set. For more details, please see (*24*).

To assess the extent to which the RNA processing changes observed after infection with *Listeria* and *Salmonella* in human macrophages are a ubiquitous characteristic of mammalian response to infection, we estimated RNA processing changes in human, chimpanzee, and rhesus macaque whole blood (a mix of white blood cells, with red blood cells lysed, see 1.1) infected with lipopolysaccharide (LPS). Using a similar study design as used previously, for each species, RNA from 6 samples were sequenced at both 2 hours and 24 hours after infection, paired with matched controls. For each RNA processing event type, events were used for further downstream analyses if they were detected in both non-infected and infected condition in 100% of samples at both timepoints. For each sample a percent spliced in (PSI) value was calculated for each event. Differential RNA processing was estimated using MISO by pairwise

comparisons of non-infected and infected samples (isng the matched non-infected sample from the corresponding timepoint) to calculate a $\Delta\Psi$ value and a BF for each sample-condition-timepoint condition. Events were considered to be significantly changing after infection if at least 50% (n $\geq$ 3) of samples had a $BF \geq 5$ and the $|mean\Delta\Psi| \geq 0.05$ (Table S9).

One concern was that the transcript annotations for chimpanzee were quite poor, relative to the well-annotated human and macaque transcriptomes. This resulted in a paucity of TandemUTR events detected across all samples in chimpanzee. Thus, for chimpanzee TandemUTRs, we used events that were detected in at least 1 individual, regardless of timepoint, in order to have the power to detect a sufficient number of significantly changing TandemUTRs.

1.3.6 Inter-individual analyses

Candidate *trans*-regulatory factors were identified by correlating variation in genome-wide RNA processing shifts and variation in the fold changes of gene expression levels across individuals. An individual-specific genome-wide RNA processing shift was measured by taking the mean $\Delta\Psi$ across all events for each RNA processing category. For each gene expressed in either non-infected or infected conditions, we calculated a Spearman correlation between the fold changes in gene expression and the individual-specific mean $\Delta\Psi$ values. To assess significance, we performed a 1000 permutations by scrambling the individual labels and assigned a p-value based on frequency of a more extreme correlation value occuring in the permuted data relative to the observed correlation value. P-values were then corrected by the Benjamini-Hochberg method to arrive at an FDR. Functional categories for known RNA binding proteins were assigned based on gene ontology annotations.

1.3.7 Enrichment of splicing regulatory elements

To search for splicing regulatory elements we considered kmers of length 6 that matched mammalian motifs identified by the following sources: exonic splicing enhancers (ESEs) from Fairbrother et al. 2002 (*25*), exonic splicing silencers (ESSs) from Wang et al. 2004 (*26*), intronic splicing enhancers (ISEs) from Wang et al. 2012 (*27*), and intronic splicing silencers (ISSs) from Wang and Wang 2014 (*28*). For each skipped exon, we used both (1) the entire exon to search for ESEs and ESSs or (2) the 100nt of intronic sequence upstream and downstream of each exon to search for ISEs and ISSs. For each category of SREs, an enrichment score was calculated as follows:

$$\textit{fold enrichment} = \frac{\sum kmers_{SRE} / \sum kmers_{total} \textit{ in significant SEs}}{\sum kmers_{SRE} / \sum kmers_{total} \textit{ in non-significant SEs}}$$

Significance of each enrichment was assessed using a *Fisher-exact test*.

1.3.8 Gene Ontology analyses

To test for enrichment of functional annotations among genes differently expressed upon infection we performed a Gene Set Enrichment Analyses (GSEA) on a list of genes ranked by p-values, as implemented in the GOrilla software tool (*29*).For all other GO-enrichment analyses we used a custom script to test for enrichment of functional annotations among genes significantly changing splicing in response to infection, in order to avoid significant gene ontology terms with overlapping gene sets. Specifically, the script uses the gene ontology annotation databases downloaded from the Gene Ontology Consortium website (*30*) and searches for the gene ontology term with most significant enrichment of significantly changing splicing changes relative to a background of all expressed genes with annotated splicing events of the same event type. Genes that belong to the most significant gene ontology term are then removed from the foreground and background sets of genes and the process is repeated iteratively until no genes are left in the foreground set. P-values are computed using a *Fisher-exact test* and then corrected using a Benjamini-Hochberg multiple test correction. For the gene ontology representations in Figure 2B and Figure S9A, we used the REVIGO web interface (*31*) to summarize the significant gene ontology terms ($FDR \leq 10\%$) into super-clustered of gene ontology terms.

1.3.9 Analysis of 3'RNA-seq data

Smart-3Seq (3'RNA-seq) reads were processed using in-house scripts. First, we processed the reads to trim unique molecular identifiers (UMI) and polyA sequences from the ends of reads, and retain only processed reads that are ¿20bp. Reads are then mapped using TopHat2 (*4*), with the fr-secondstrand option. Mapped reads are filtered to remove PCR duplicates and sort by UMI sequences. Mapped and processed reads that fall within Tandem 3' UTR regions were used to make a meta-gene plot of read density (normalized by million mapped reads) with a smoothing window of 10bp. Reads in individual 3' UTRs were used to calculate a $\Delta\Psi$ statistic, where Ψ is defined as:

$$\Psi = \frac{\sum 3'reads_{extended}}{\sum 3'reads_{core} + \sum 3'reads_{extended}}$$

1.3.10 Analysis of miRNA-seq data

After demultiplexing, sequences matching the $3'$ adaptor sequence were identified and trimmed. A minimum matching of the 6 first bases of the adaptor sequence was required, following which we discarded all reads shorter than 16 or longer than 26 bases, corresponding to the known length distribution of mammalian miRNAs. After these filtering steps, we obtained an average of 5.8 million (minimum 3.1 million) clean, short reads per sample that were used for small RNA quantification (Table S10).

miRNAs quantification. Sequences were aligned to the human reference genome (build GRCh37/hg19) using bowtie (v0.12.7) (*32*) allowing for 2 mismatches (-v 2) and reported all best alignments for reads that mapped equally well to more than one genomic location (-a best strata). We suppressed reads with more than 50 possible alignments (-m 50). On average, 96% of reads aligned to the human genome, of which 60% aligned uniquely. The remaining 40% of reads had more than one best alignment, which is expected given that miRNAs are short and tend to occur in families that share highly similar sequences. To avoid cross-mapping artefacts, we used a correction strategy that assigns weights to each of the candidate mapping loci of multiply aligning reads, as we have described previously (*33*).

miRNA differential expression We used DESeq2 (*8*) to identify differentially expressed miRNAs upon infection. Specifically, for each experimental condition we compared miRNA expression levels between non-infected and infected samples at the same time point limiting the analyses to miRNAs having an average expression higher than 10 reads in at least one of the experimental conditions. In order to increase power to detect differentially expressed miRNAs we explored the paired nature of our data by specifying donor identity in our model. We corrected for multiple testing using the Benjamini-Hochberg FDR method (Table S10).

Enrichment of miRNA target sites. All predicted miRNA target sites within annotated TandemUTR core and core+extended regions were obtained using TargetScan (v6.2) (*34*). Further analyses used target sites matching the following set of criteria: (i) the miRNA was expressed (corrected count $\geq$ 4) in either the non-infected or infected condition, (ii) the TandemUTR region was expressed and met the criteria outlined in Supplementary Section 1.3.5, and (3) there was at least 1 target site for a given miRNA in the extended regions of expressed TandemUTRs. Following all of these selection criteria, we considered the target sites for 163 miRNAs in *Listeria* and 161 miRNAs in *Salmonella*. Target site counts per region were normalized by the length of the region to get the density of miRNA target sites. For each miRNA, we calculated the fold enrichment of target site densities as follows:

$$\textit{fold enrichment} = \frac{mean(x_i)\,\textit{for significant TandemUTRs}}{mean(x_i)\,\textit{for non-significant TandemUTRs}},$$

where x_i = target site density in the extended region of a 3' UTR for miRNA i

The non-significant Tandem UTRs for the background distribution were chosen by matching the distribution of relative GC contents ($\%GC_{core}/\%GC_{extended}$) displayed by significant TandemUTRs in order to account for possible biases due to differential sequence compositions. The relative GC contents were chosen since the enrichment score inherently tests for the enrichment in the extended region relative to the core region per 3' UTR. Significance of each fold enrichment was assessed using a *Mann-Whitney U test* between the mean density of target sites for non-significant and significant UTRs. P-values for multiple testing using the Benjamini-Hochberg FDR method.

References and Notes

1. Beck AH, Weng Z, Witten DM, Zhu S, Foley JW, Lacroute P, Smith CL, Tibshirani R, van de Rign M, Sidow A, West RB. (2010) 3'-End Sequencing for Expression Quantification (3SEQ) from Archival Tumor Samples. *PLOS One* 5(1):e8768.

2. Zhu YY, Machleder EM, Chenchik A, Siebert PD. (2001) Reverse transcriptase template switching: a SMART approach for full-length cDNA library construction. *Biotechniques* 30(4):892-7.

3. Berg MG, Singh LN, Younis I, Liu Q, Pinto AM, Kaida D, Zhang Z, Cho S, Sherrill-Mix S, Wan L, Dreyfuss G. (2012) U1 snRNP Determines mRNA Length and Regulates Isoform Expression. *Cell* 150(1):53-64.

4. Kim D, Pertea G, Trapnell C, Pimentel H, Kelley R, Salzberg SL (2013) TopHat2: accurate alignment of transcriptomes in the precense of insertions, deletions and gene fusions. *Genome Biology* 14:R36.

5. Liao Y, Smyth GK, Shi W (2014) featureCounts: an efficient general purpose program for assigning sequence reads to genomic features. *Bioinformatics* 30(7):923-30.

6. Li B, Dewey CN (2011) RSEM: accurate transcript quantification from RNA-Seq data with or without a reference genome. *BMC Bioinformatics* 12:323.

7. Bray N, Pimentel H, Melsted P, Pachter L (2015) Near-optimal RNA-Seq quantification. *arXiv*

8. Love MI, Huber W, Anders S (2014) Moderated estimation of fold change and dispersion for RNA-seq data with DESeq2. *Genome Biology* 15:550.

9. Welch B-L (1947) The generalization of student problem when several different population variances are involved. *Biometrika* 34:28-35.

10. Aitchison J (1982) The Statistical Analysis of Compositional Data. *Journal of the Royal Statistical Society. Series B (Methodological)* 44(2):139-177.

11. Egozcue J-J, Pawlowsky-Glahn V, Mateu-Figueras G, Barcel-Vidal C (2003) Isometric logratio transformations for compositional data analysis. *Mathematical Geology* 35(3):279-300.

12. Aitchison J (1986) The statistical analysis of compositional data. Monographs on statistics and applied Probability. *Chapman* & *Hall, London*.

13. Niu L, Huang W, Umbach D-M, Li L (2014) IUTA: a tool for effectively detecting differential isoform usage from RNA-Seq data. *BMC Genomics* 15(1):862-875.

14. Krishnamoorthy K, Yu J (2004) Modified Nel and Van der Merwe test for the multivariate BehrensFisher problem. *Statistics & probability letters* 66(2):161-169.

15. Srivastava M-S, Katayama S, Kano Y (2013) A two sample test in high dimensional data. *Journal of Multivariate Analysis* 114:349-358.

16. Chen S-X, Qin Y-L (2010) A two-sample test for high-dimensional data with applications to gene-set testing. *The Annals of Statistics* 38(2):808-835.

17. Hurlbert S (1971) The nonconcept of diversity: a critique and alternative parameters. *Ecology* 52:577-586.

18. Shannon C-E, Weaver W (1963) The mathematical theory of communication. *University of Illinois Press, Urbana*.

19. Simpson E-H (1949) Measurement of diversity. *Nature* 163:688.

20. Katz Y, Wang ET, Airoldi EM, Burge CB. (2010) Analysis and design of RNA sequencing experiments for identifying isoform regulation. *Nature Methods* 7:1009-1015.

21. Dobin A, Davis CA, Schlesinger F, Drewndow J, Zaleski C, Jha S, Batut P, Chaisson M, Gingeras TR. (2012) STAR: ultrafast universal RNA-seq aligner. *Bioinformatics* 29:15-21.

22. Trapnell C, Roberts A, Goff L, Pertea G, Kim D, Kelley DR, Pimentel H, Salzberg SL, Rinn JL, Pachter L. (2012) Differential gene and transcript expression analysis of RNA-seq experiments with TopHat and Cufflinks. *Nature Protocols* 7:562-578.

23. Barbosa-Morais N, Irimia M, Pan Q, Ziong HY, Gueroussov S, Lee LJ, Slobodeniuc V, Kutter C, Watt S, Colak R, Kim TH, Misquitta-Ali CM, Wilson MD, Kim PM, Odom DT, Frey BJ, Blencowe BJ. (2012) The evolutionary landscape of alternative splicing in vertebrate species. *Science* 338:1587-1593.

24. Merkin J, Russell C, Chen P, Burge CB. (2012) Evolutionary Dynamics of Gene and Isoform Regulation in Mammalian Tissues. *Science* 338:1593-1599.

25. Fairbrother WG, Yeh RF, Sharp PA, Burge CB (2002) Predictive identification of exonic splicing enhancers in human genes. *Science* 297:1007-1013.

26. Wang Z, Rolish ME, Yeo G, Tung V, Mawson M, Burge CB. (2004) Systematic Identification and Analysis of Exonic Splicing Silencers. *Cell* 119(6):831-845.

27. Wang Y, Ma M, Xiao X, Wang Z (2012) Intronic splicing enhancers, cognate splicing factors and context-dependent regulation rules. *Nature Structural & Molecular Biology* 19:1044-1052.

28. Wang Y, Wang Z (2014) Systematical identification of splicing regulatory cis-elements and cognate trans-factors. *Methods* 65:350-358.

29. Eden E, Navon R, Steinfeld I, Lipson D, Yakhini Z (2009) GOrilla: a tool for discovery and visualization of enriched GO terms in ranked gene lists. *BMC bioinformatics* 10:48.

30. The Gene Ontology Consortium (2015) Gene Ontology Consortium: going forward. *Nucleic Acids Research* 43:D1049-D1056.

31. Supek F, Bosnjak M, Skunca N, Smuc T (2011) REVIGO summarizes and visualizes long lists of Gene Ontology terms. *PLoS One* 6(7):e21800.

32. Langmead B, Trapnell C, Pop M, Salzberg SL (2009) Ultrafast and memory-efficient alignment of short DNA sequences to the human genome. *Genome Biology* 10:R25.

33. Siddle KL, Deschamps M, Tailleux L, Nedelec Y, Pothlichet J, Lugo-Villarino G, Libri V, Gicquel B, Neyrolles O, Laval G, Patin E, Barreiro LB, Quintana-Murci L (2014) A genomic portrait of the genetic architecture and regulatory impact of microRNA expression in response to infection. *Genome Research* 24:850-859.

34. Friedman RC, Farh KK, Burge CB, Bartel DP (2009) Most Mammalian mRNAs Are Conserved Targets of MicroRNAs. *Genome Research* 19:92-105.

35. DeLuca DS, Levin JZ, Sivachenko A, Fennell T, Nazaire MD, Williams C, Reich M, Winckler W, Getz G (2012) RNA-seq metrics for quality control and process optimization. *Bioinformatics* 28(11):1530-1532.

2 Supplementary Figures

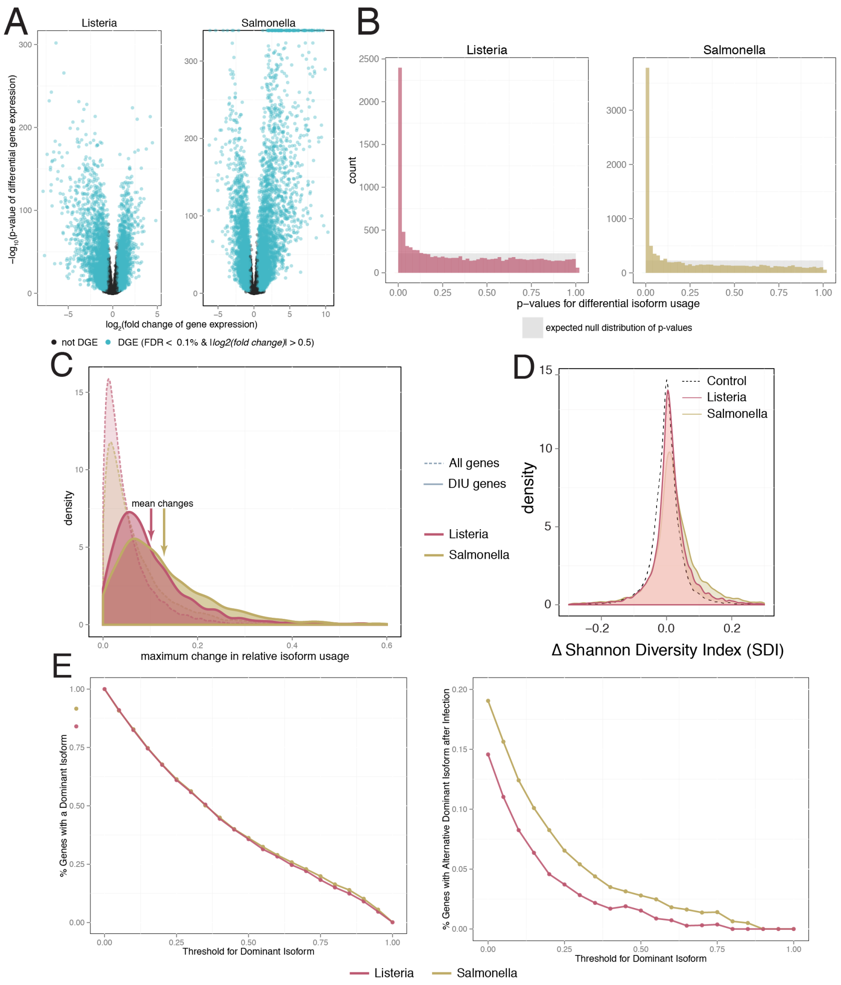

Figure S1: **Differential isoform usage after infection.** (A) Volcano plots of differential expressionafter infection with *Listeria* and *Salmonella* in the left and right panels, respectively. $-log_{10}$ p-values (*y-axis*) testing for differential expression are plotted against average log_2 fold changes in expression levels (*x-axis*) for genes that are not differentially expressed (*black*) and genes that with significant differential expression after infection ($FDR \leq 0.1\%$ and $|log_2(\text{fold change})| \geq 0.5$; *blue*). (B) Distribution of p-values for the differential isoform usage test upon infection with *Listeria* and *Salmonella*. Expected distribution of p-values under the null hypothesis of no significant difference between the mean relative isoform usage is shown in grey. (C) A comparison of DIU effect sizes between DIU genes at 1% FDR (following infection with *Listeria* in *dark pink* and *Salmonella* in *gold*) and the background of all genes (*lighter colors*). Effect sizes are defined as maximum change in relative isoform abundances per gene upon infection. (D) Distributions of Shannon diversity index (SDI) after infection with *Listeria* and *Salmonella*. Null distribution (*black dotted line*) was generated by permuting samples across conditions. (E) The percentage of genes with a dominant isoform before infection (*left*) and the fraction of these genes where the dominant isoform changes after infection (*right*).

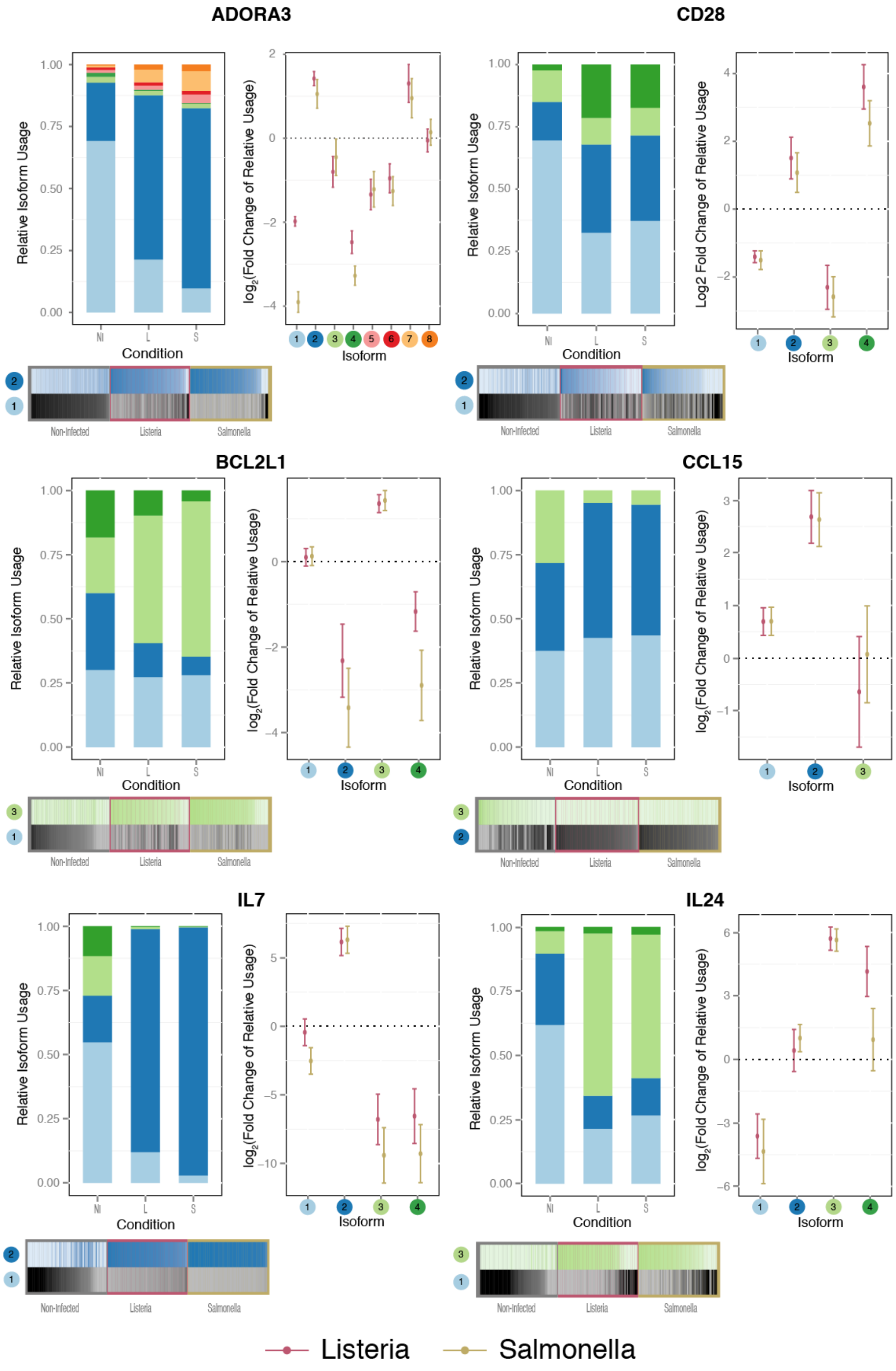

Figure S2: **Representative examples of immune-related genes with significant DIU after infection.** Heatmaps below each example represent the variation in isoform usage across the 60 individuals, where each vertical bar represents one individual. The dominant isoform in non-infected samples is represented in emphgrey, while the predominant isoform in infected samples is in a color. Darker bars represent increased relative usage, while lighter bars represent decreased relative usage.

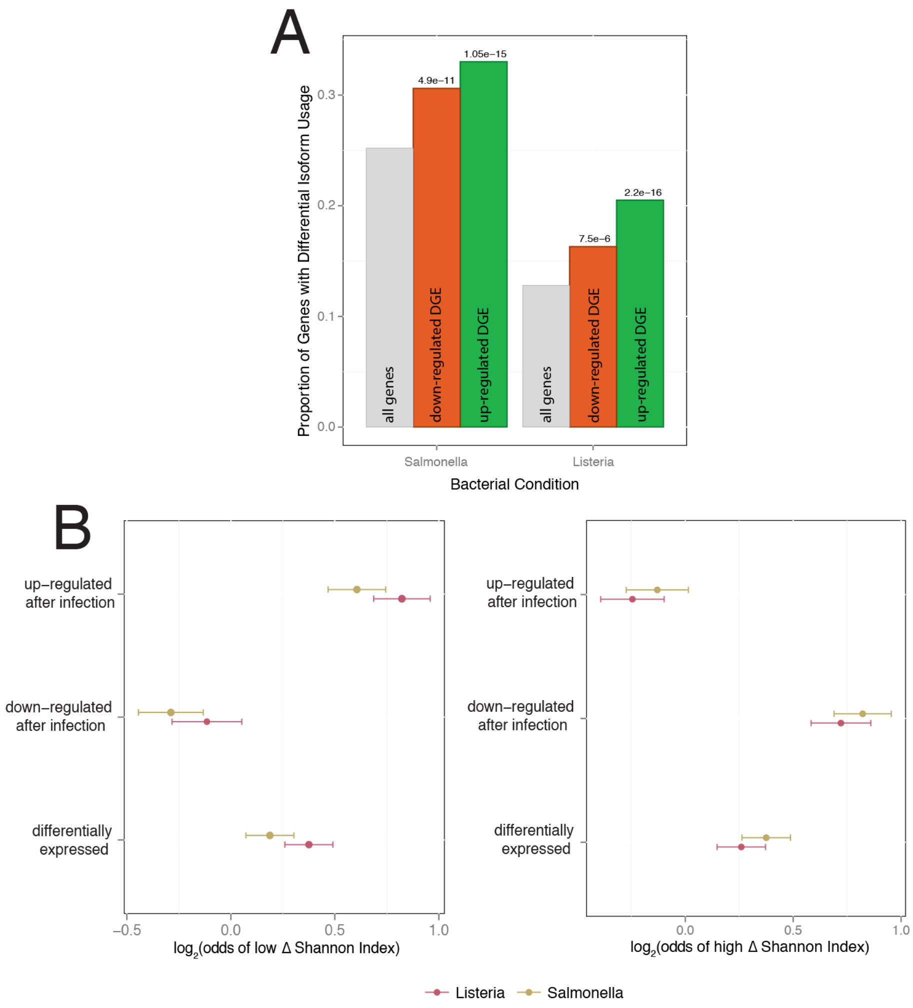

Figure S3: **Gene expression and differential isoform diversity after infection.** (A) Proportion of genes with differential isoform usage (*y-axis*) among all genes (*grey*), differentially expressed genes that are down-regulated after infection (*orange*), and differentially expressed genes that are up-regulated after infection (*green*). (B) log_2 fold changes (with standard error bars) for odds of low $\Delta_{Shannon}$ and high $\Delta_{Shannon}$ among the set of all the differentially expressed genes, up-regulated differentially expressed genes, and down-regulated differentially expressed genes, following infection with *Listeria* (*dark pink*) and *Salmonella* (*gold*).

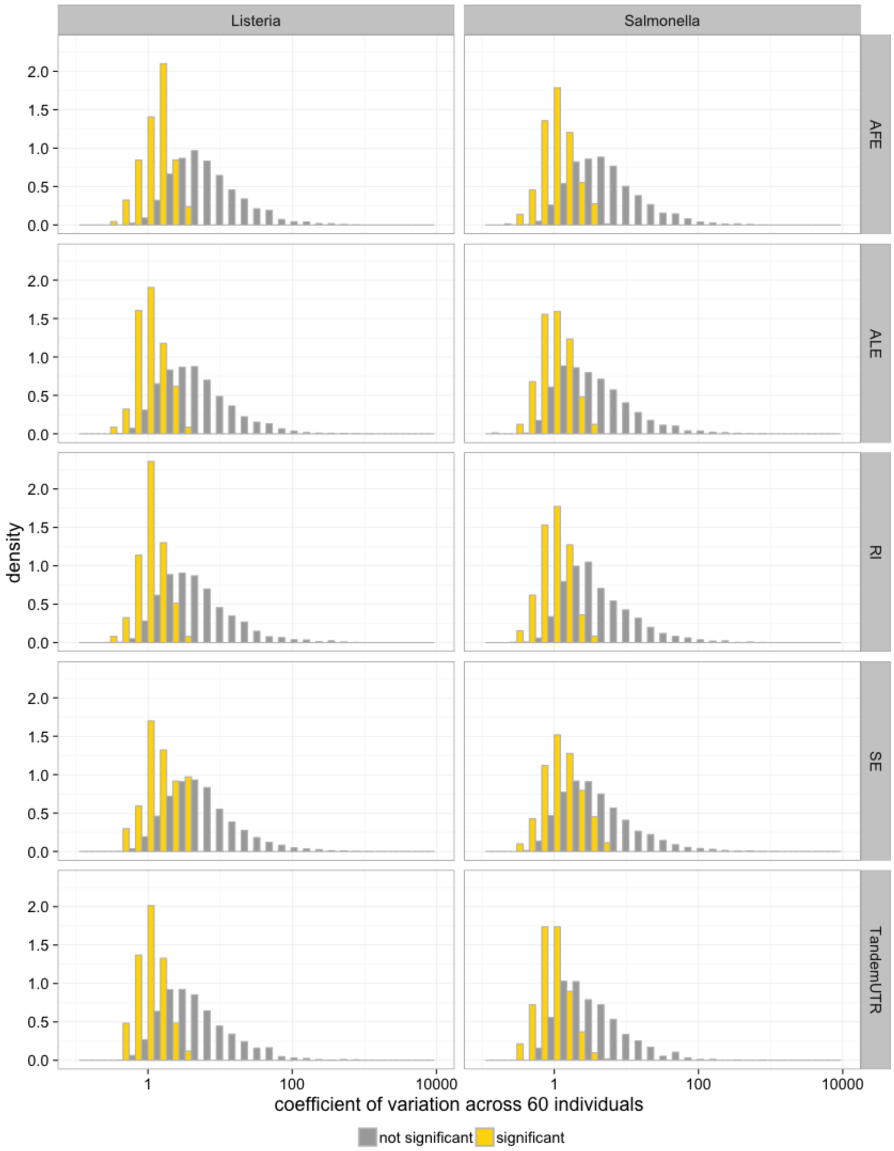

Figure S4: **Variance in RNA processing across 60 individuals.** Distribution of coefficient of variation (*x-axis*) in $\Delta\Psi$ values for each isoform in a given RNA processing category.

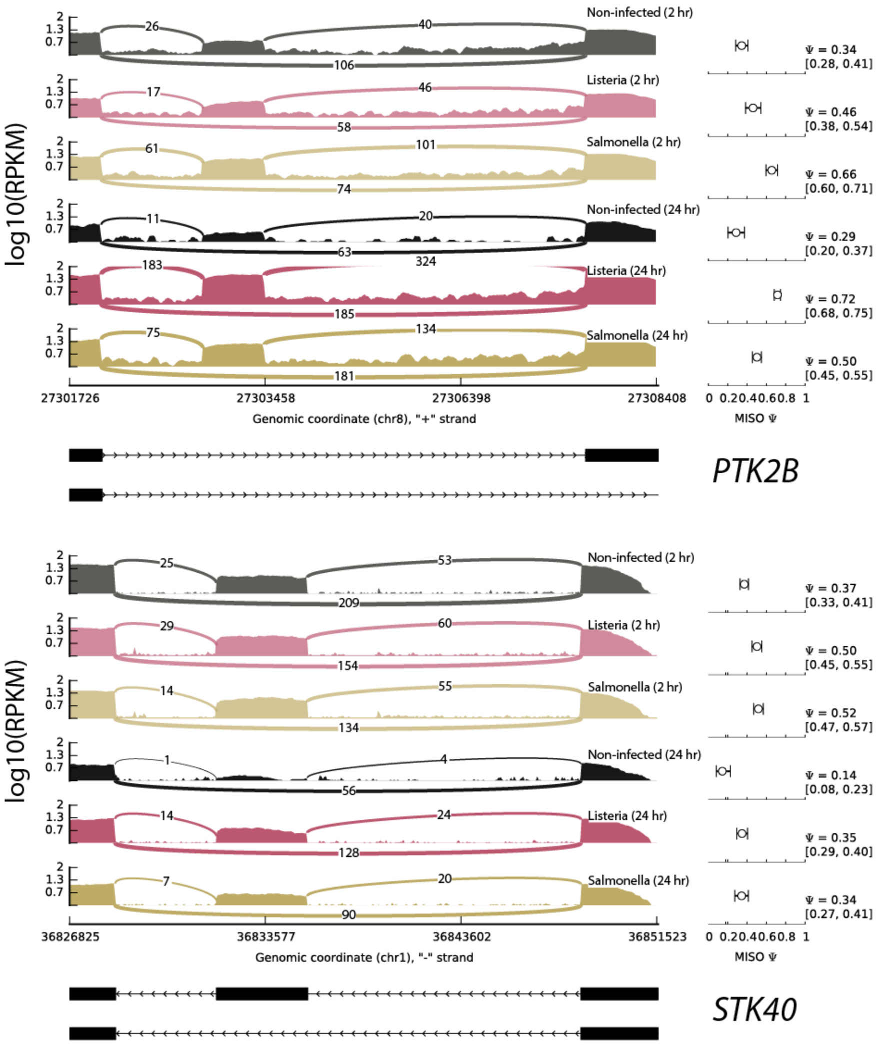

Figure S5: **Two representative examples of genes with significant skipped exon changes after infection.** Sashimi plots for two genes, *PTK2B* and *STK40*, that have significant changes in skipped exon usage both after 2 hours and 24 hours of infection with either bacteria.

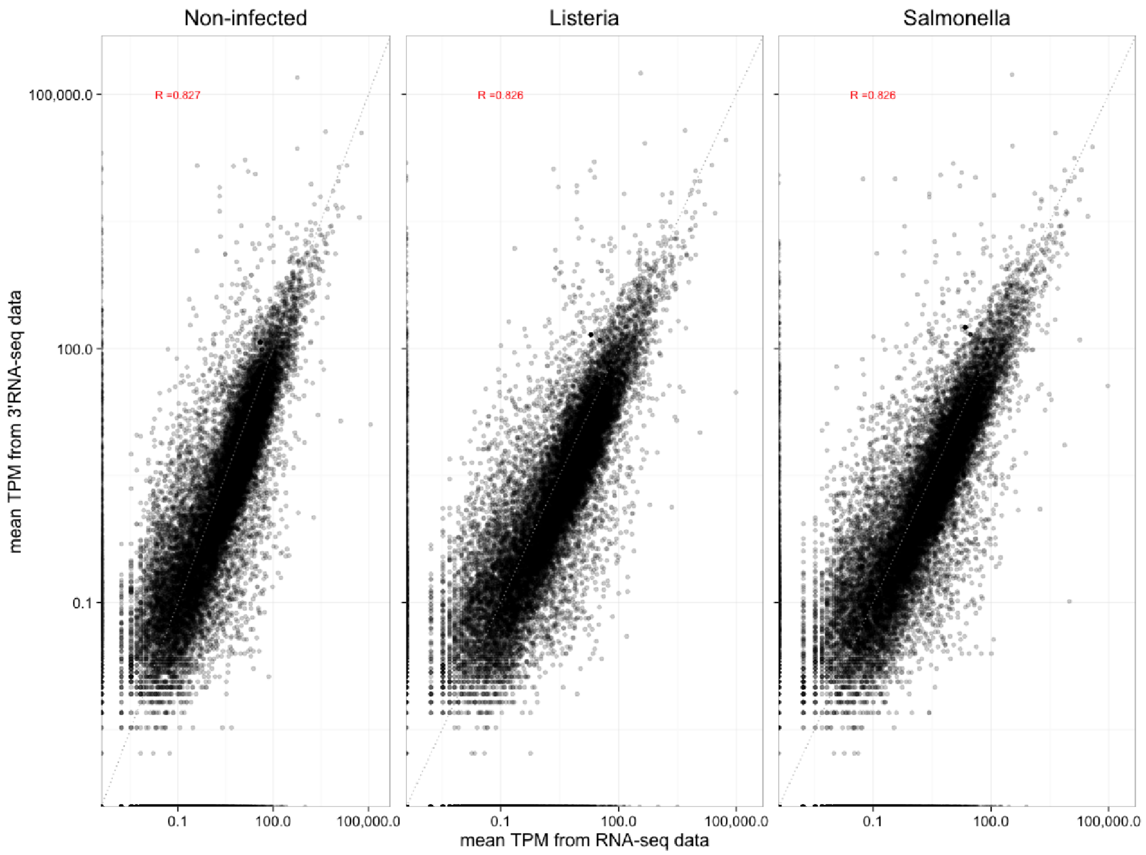

Figure S6: **Relationship between 3'RNA-seq and RNA-seq data.** Correlations between TPMs from 3' RNA-seq data (*y-axis*) and TPMs from RNA-seq data (*x-axis*).

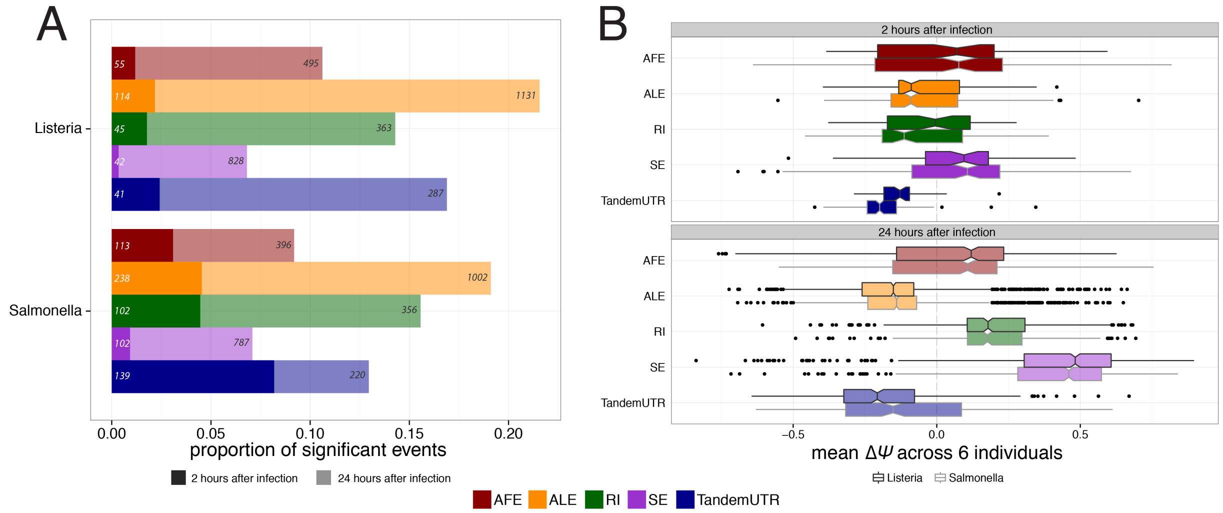

Figure S7: **RNA processing changes 24hr after infection.** (A) Proportion of significantly changing events (*x-axis*) after 2 hours of infection (*dark colors*) and 24 hours of infection (*light colors*) with either *Listeria* (*top*) or *Salmonella* (*bottom*). Numbers indicate the significant events at corresponding timepoints using only the 6 individuals used for these cross-timepoint analyses. (B) Distribution of $\Delta\Psi$ values (*x-axis*) for significantly changing events in each event type after either 2 hours (*top*) or 24 hours (*bottom*) of infection.

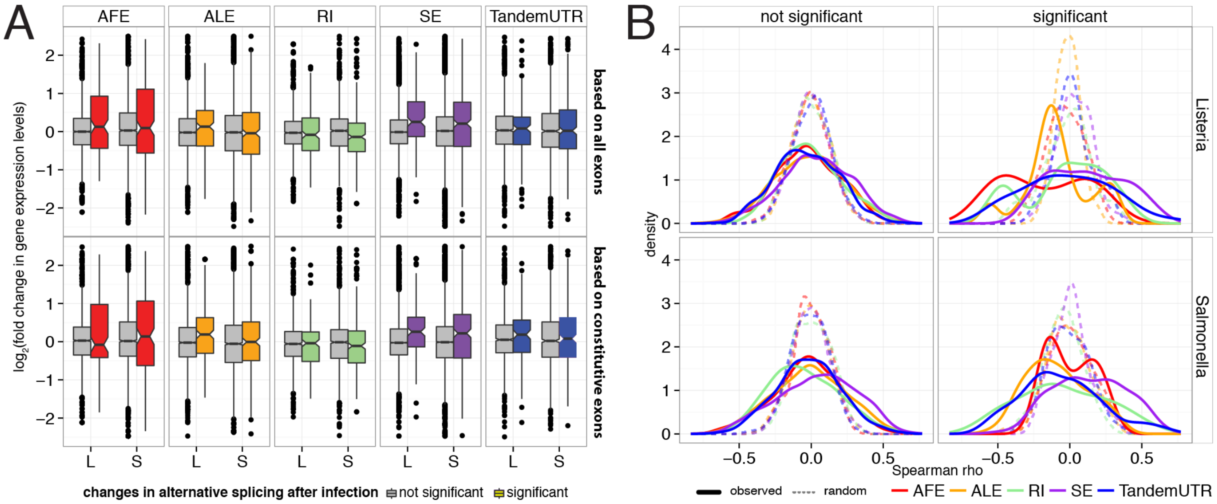

Figure S8: **Relationships between RNA processing and gene expression changes after infection.** (A) Distributions of overall fold changes in gene expression (*y-axis*, log_2 scale) for genes that have significant splicing changes in each event type (*colored boxplots*) and genes with no splicing changes after infection (*grey*). Gene expression values are calculated using either full transcript models (*top*) or only constitutively included exons (*bottom*). (B) Distribution of spearman correlations between the $\Delta\Psi$ of an event and the fold change in gene expression, across individuals, for events that are not significantly changing after infection (*left*) and significantly changing events (*right*). Solid lines represent the observed values and dotted lines represent a distribution of correlations after permuting the correspondence between $\Delta\Psi$s and fold changes in gene expression.

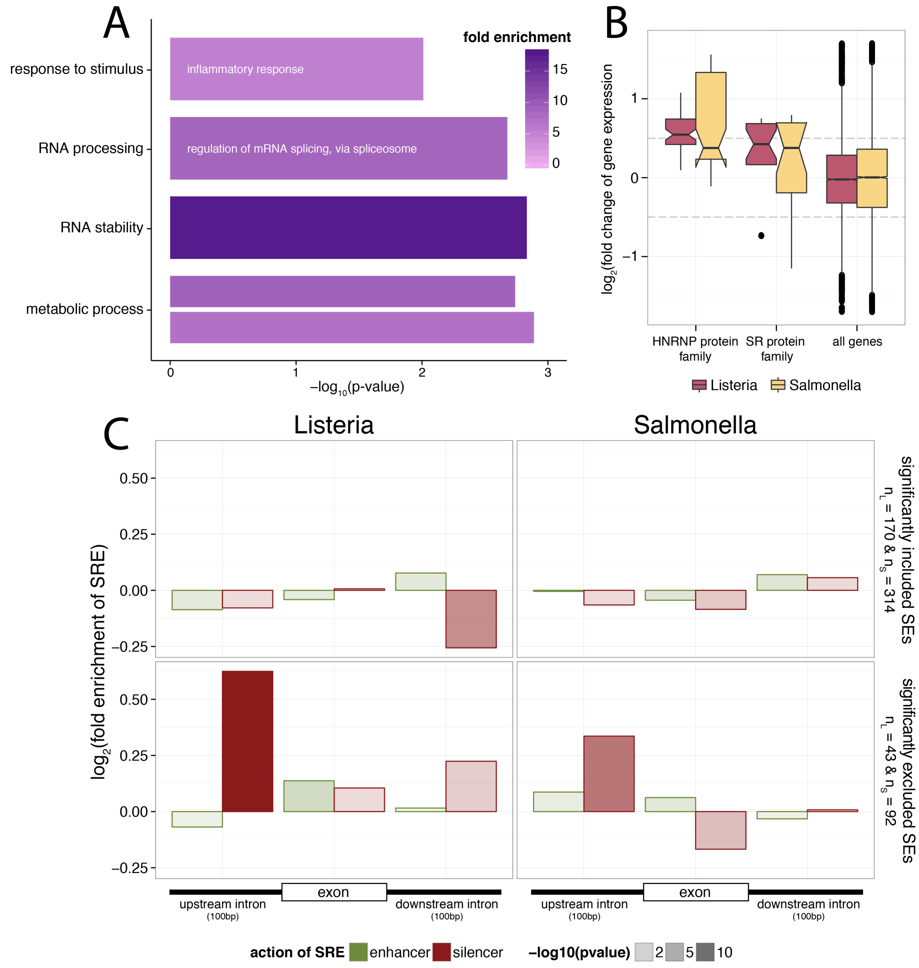

Figure S9: **Characteristics of significantly changing skipped exon events.** (A) Significantly enriched gene ontology categories for genes with significant skipped exon changes after infection. Color indicates the fold enrichment of the number of observed genes relative to the number of genes expected to be in that category. (B) Fold changes in gene expression (*y-axis*, log_2 scale) after infection for 2 families of splicing factors (hnRNPs and SR proteins) relative to a background distribution of fold changes in all genes (C) Fold enrichments of splicing regulatory elements (SREs) in exonic regions and surrounding intronic regions ($\pm$ 100bp). SREs assessed included exonic splicing enhancers (*green* in exons), exonic splicing silencer (*red* in exons), intronic splicing enhancers (*green* in introns), and intronic splicing silencers (*green* in introns). Enrichments were calculated separately for significantly included skipped exons (*top*) and significantly excluded skipped exons (*bottom*). Shading of the bars indicates the significance of the enrichment.

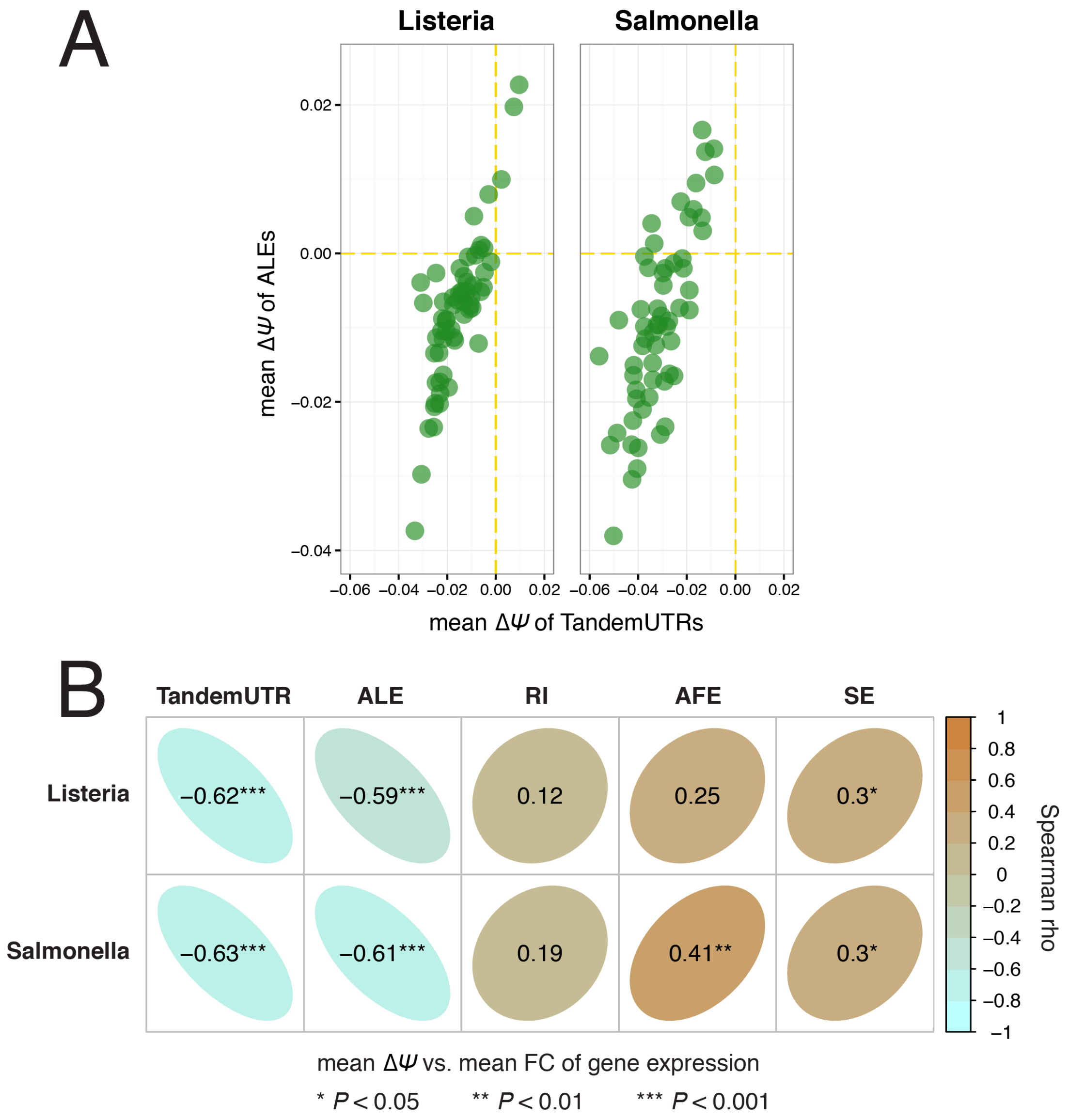

Figure S10: **Inter-individual variation of global shifts in alternative splicing.** (A) Correlations between the mean $\Delta\Psi$ values per individual for ALEs (*x-axis*) and TandemUTRs (*y-axis*). (B) Spearman correlations between the mean $\Delta\Psi$ value per individual and the mean fold change of gene expression per individual for corresponding genes.

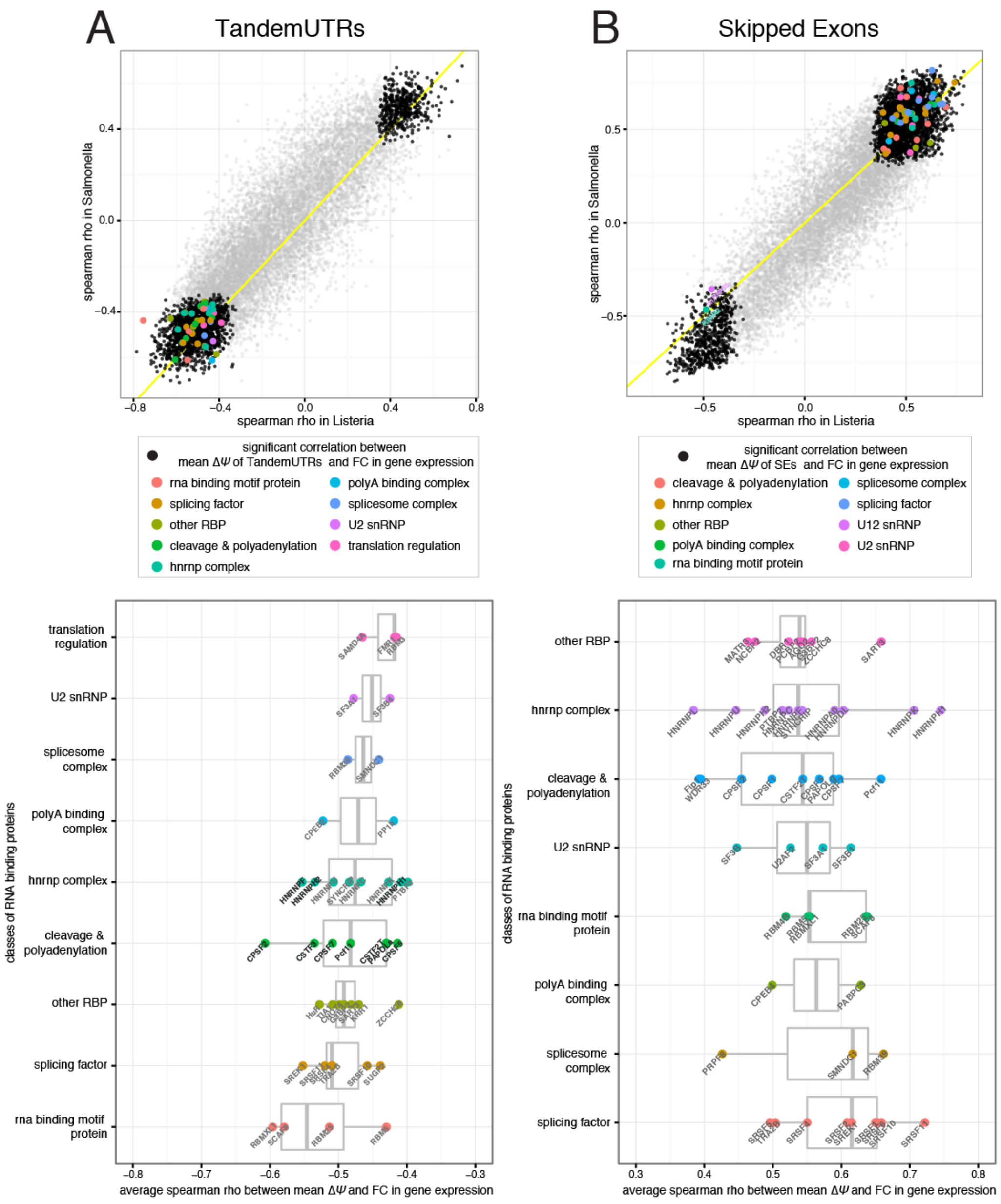

Figure S11: **Identifying RNA binding factors that might be involved in *trans*-regulation of RNA processing after infection.** For both TandemUTRs (A) and Skipped exons (B), the top panel is a scatter plot of Spearman correlations between the individual-specific mean $\Delta\Psi$ values and individual-specific fold change of gene expression values for all expressed genes (*grey*) in both *Listeria* (*x-axis*) and *Salmonella* (*y-axis*). Genes with significant correlations ($FDR \leq 1\%$) in both *Listeria* and *Salmonella* conditions are plotted in black, and those factors with known RNA-binding properties are colored by their functional category. The bottom panels show distributions of the average Spearman correlation values for each of the RNA-binding functional categories with significant correlations.

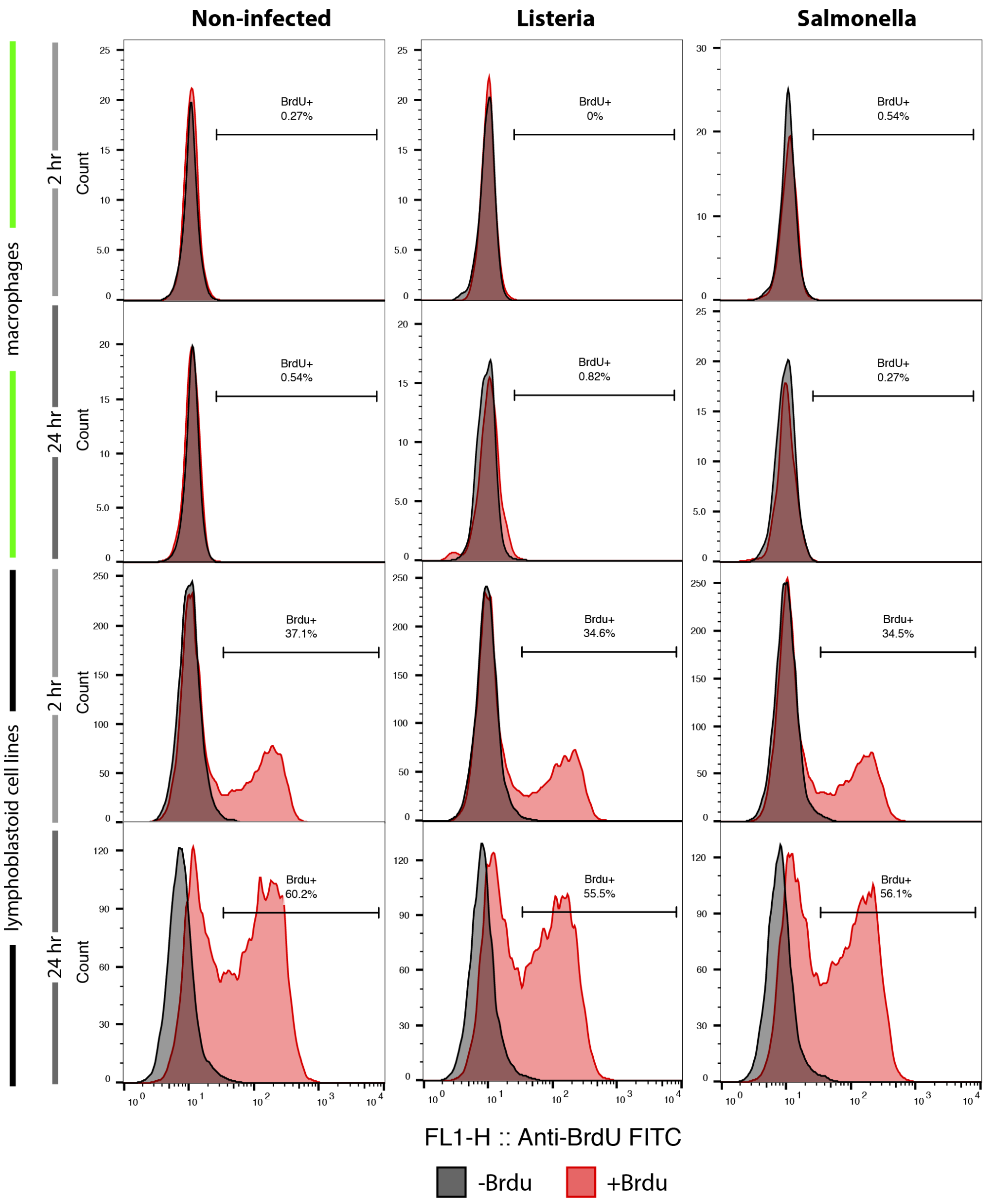

Figure S12: **Cellular proliferation after bacterial infection.** BrdU cell proliferation assay in macrophages (top panel) and LCLs (bottom panel) in non-infected cells and in cells infected with *Listeria* or *Salmonella* for both 2 and 24 hours. BrdU incorporates into newly synthesized DNA and therefore the quantity of BrdU incorporated into cells (*x-axis*) is a direct indication of cell proliferation. No evidence for cellular proliferation was observed in macrophages, in contrast to the high rates of proliferating cells observed in our positive control population of LCLs.

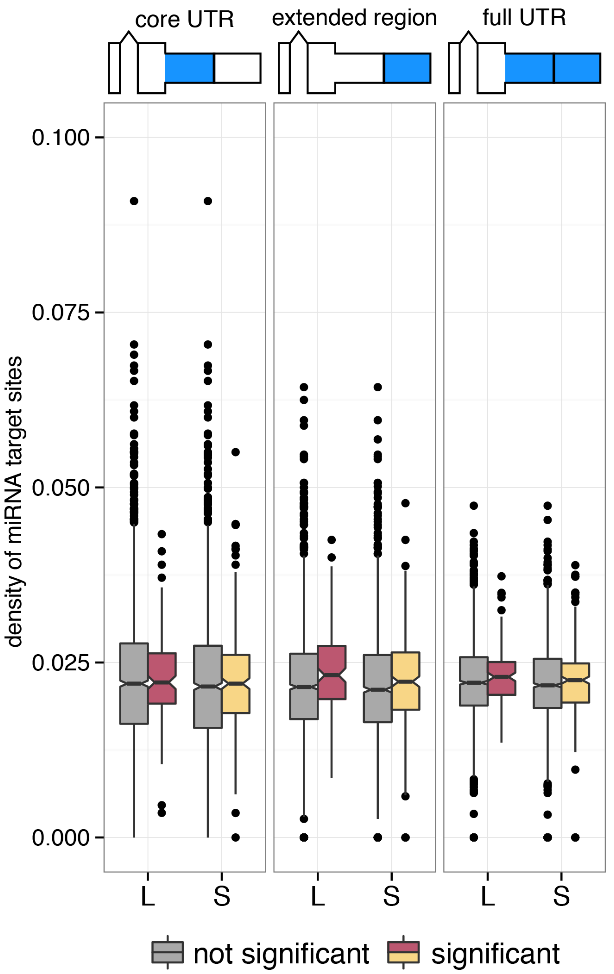

Figure S13: **Density of miRNA target sites in 3′ UTR regions.** Barplots in grey indicate Tandem 3′ UTRs that are not changing after infection, while colored barplots indicate Tandem 3′ UTRs that are significantly changing after either *Listeria* (*pink*) or *Salmonella* (*yellow*) infections.

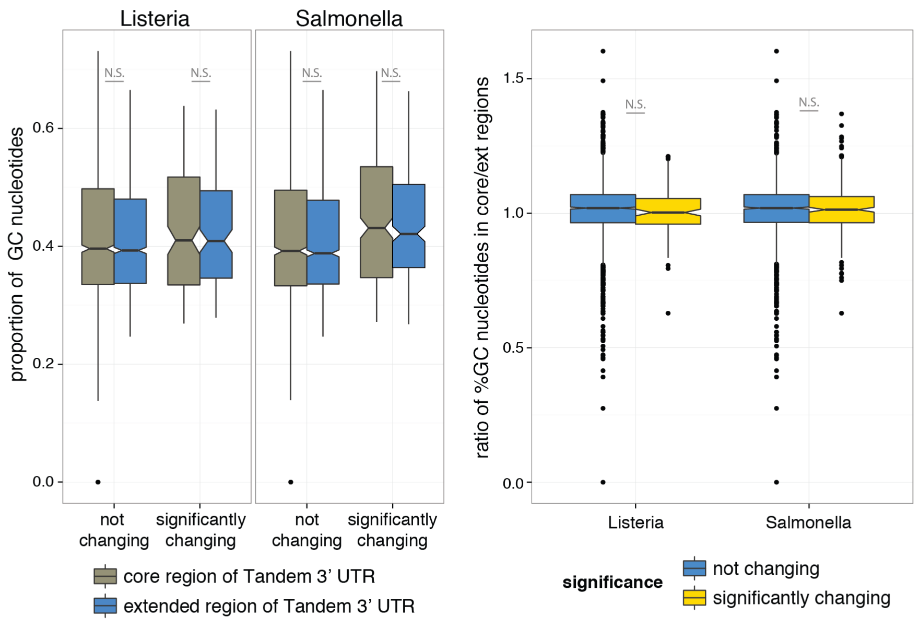

Figure S14: **Nucleotide composition of Tandem 3' UTR regions.** The distribution of GC content in core (*brown*) and extended (*blue*) regions of Tandem 3' UTRs that are either not changing or significantly changing after infection (*left panel*). While there 3' UTRs that are significantly changing generally have greater overall GC content, this is true for both the core and extended regions of the 3' UTRs. Thus, the distributions of relative GC content when comparing the regions is distributed around 1 for both significantly changing (*yellow*) and not changing (*blue*) Tandem 3' UTRs (*right panel*).

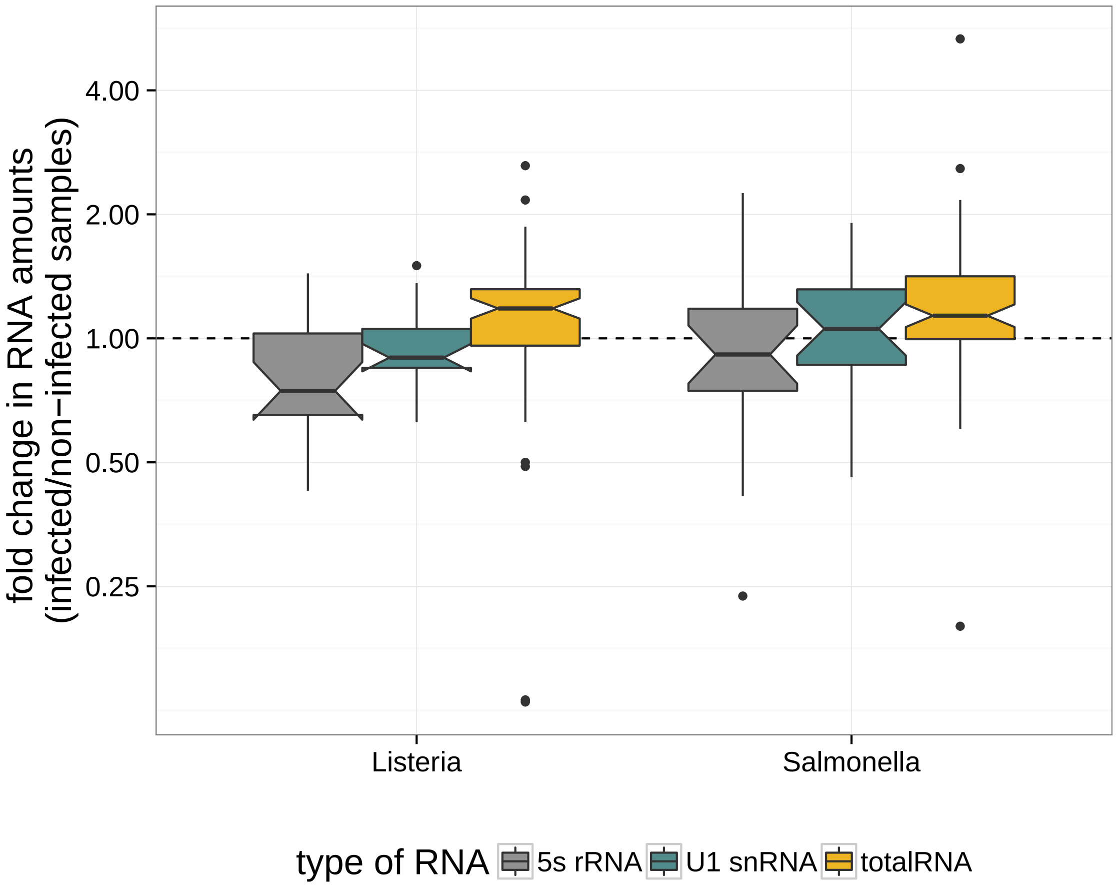

Figure S15: **Investigating effects of telescripting.** Distributions of fold changes in 5s rRNA (*grey*, n=20), U1 (*green*, n=20), and total RNA (*yellow*, n=60) concentrations after infection. 5s rRNA concentrations were calculated by taking the ΔCT value from qPCR measurements across 20 samples. Relative U1 snRNA concentrations were calculated by taking the $\Delta\Delta$CT values from qPCR measurements across 20 samples, where U1 snRNA is measured relative to 5s rRNA concentrations in the same samples. Total RNA concentrations were estimated from Nanodrop measurements of RNA extraction yields across all 60 samples in our study. All samples (both non-infected and infected) were plated at exactly the same macrophage cellular density at the start of the experiment and we have confirmed that these macrophages do not proliferate either before or after infection (Figure S12). There are no significant shifts in the distribution of 5s rRNA or U1 snRNA fold changes after infection (t-test for null of $\mu = 1$, both $P > 0.01$ for 5s rRNA and both $P > 0.2$ for U1 snRNA), while the distribution of total RNA fold changes are significantly increased after infection (t-test for null of $\mu = 1$, $P < 0.001$ for both *Listeria* and *Salmonella* samples).

3 Supplementary Table Legends

Table S1: mRNA-seq statistics. Details of RNA-seq data for samples after infection at 2 hours or 24 hours. Libraries were made as described in Supplementary Section 1.2.1.

Table S2: Gene Level Differential Gene Expression. Estimates of differential gene expression for the $14,954$ genes tested by DESeq2, as described in Supplementary Section 1.3.2. Included are the log_2 fold change estimates, p-values for differential expression, and Benjamini-Hochberg adjusted p-values for differential expression between non-infected samples and both *Listeria* and Salmonella samples.

Table S3: Gene Ontology Enrichments for Differential Expression. Enriched Gene Ontology categories for genes that are differentially expressed, or show differential isoform usage after infection. Gene Ontology analyses were performed using GSEA, as described in Supplementary Section 1.3.8.

Table S4: Differential Isoform Usage. Estimates of differential isoform usage for the $11,353$ genes tested as described in Supplementary Section 1.3.3. Included are the changes in relative isoform usage for the isoform with the greatest relative change after infection, the p-value and Benjamini-Hochberg FDR for DIU using isoform expression levels calculated using RSEM, and the FDR for DIU using isoform expression levels calculated using Kallisto.

Table S5: Change in Isoform Diversity after Infection. Estimates of Shannon diversity indicies for $11,353$ genes both before and after infection with both *Listeria* and *Salmonella*, as described in Supplementary Section 1.3.4.

Table S6: Differential isoform usage across 5 specific RNA processing categories. Estimates of Ψ and $\Delta\Psi$ values calculated by MISO (Supplementary Section 1.3.5) for each individual for each for the 5 different RNA processing categories assess in this study: alternative first exons (AFE), alternative last exons (ALE), retained introns (RI), skipped exons (SE), and Tandem 3' UTRs (TandemUTR). Also included are Bayes Factors testing for differential isoform usage per event for each individual, mean $\Delta\Psi$ values across all individuals and individuals with significant changes, and a column indicating whether an event was called significant based on the criteria defined in Supplementary Section 1.3.5.

Table S7: Summaries of Significant RNA processing categories. A summary of the proportion of annotated events that were detected and determined to be significant (according to the criteria defined in Supplementary Section 1.3.5) for each condition and RNA processing category. Included are tables for metrics after both 2 hours of infection and 24 hours of infection.

Table S8: Gene Ontology Enrichments for significant RNA processing changes. Top gene

ontology categories for genes that have significant RNA processing changes after infection with either *Listeria* or *Salmonella*. All tests were done using an iterative gene ontology analysis as described in Supplementary Section 1.3.5 and gene names refer to UniProt gene accessions.

Table S9: Summary of Significant RNA processing categories following infection with LPS, across primate species. A summary of the proportion of annotated evenst that were detected and determined to be significant (according to the criteria defined in Supplementary Section 1.3.5) for each species and RNA processing category following infection of primary whole blood cells with LPS.

Table S10: miRNA mapping and differential expression statistics. Details of short RNA-seq data for 6 samples sequenced after both 2 hours and 24 hours of infection with either *Listeria* or *Salmonella*, as described in Supplementary Sections 1.2.3 and 1.3.10. Also included are the log_2 fold change estimates and p-values for differential expression of miRNAs after both 2 hours and 24 hours of infection, tested by DESeq2 as described in Supplementary Section 1.3.10.
