# Supplementary figures and images for: Widespread Shortening of 3’ Untranslated Regions and Increased Exon Inclusion Are Evolutionarily Conserved Features of Innate Immune Responses to Infection
Source: PLoS Genet. 2016 Sep 30;12(9):e1006338. doi: 10.1371/journal.pgen.1006338 (PMC5045211; doi:10.1371/journal.pgen.1006338)

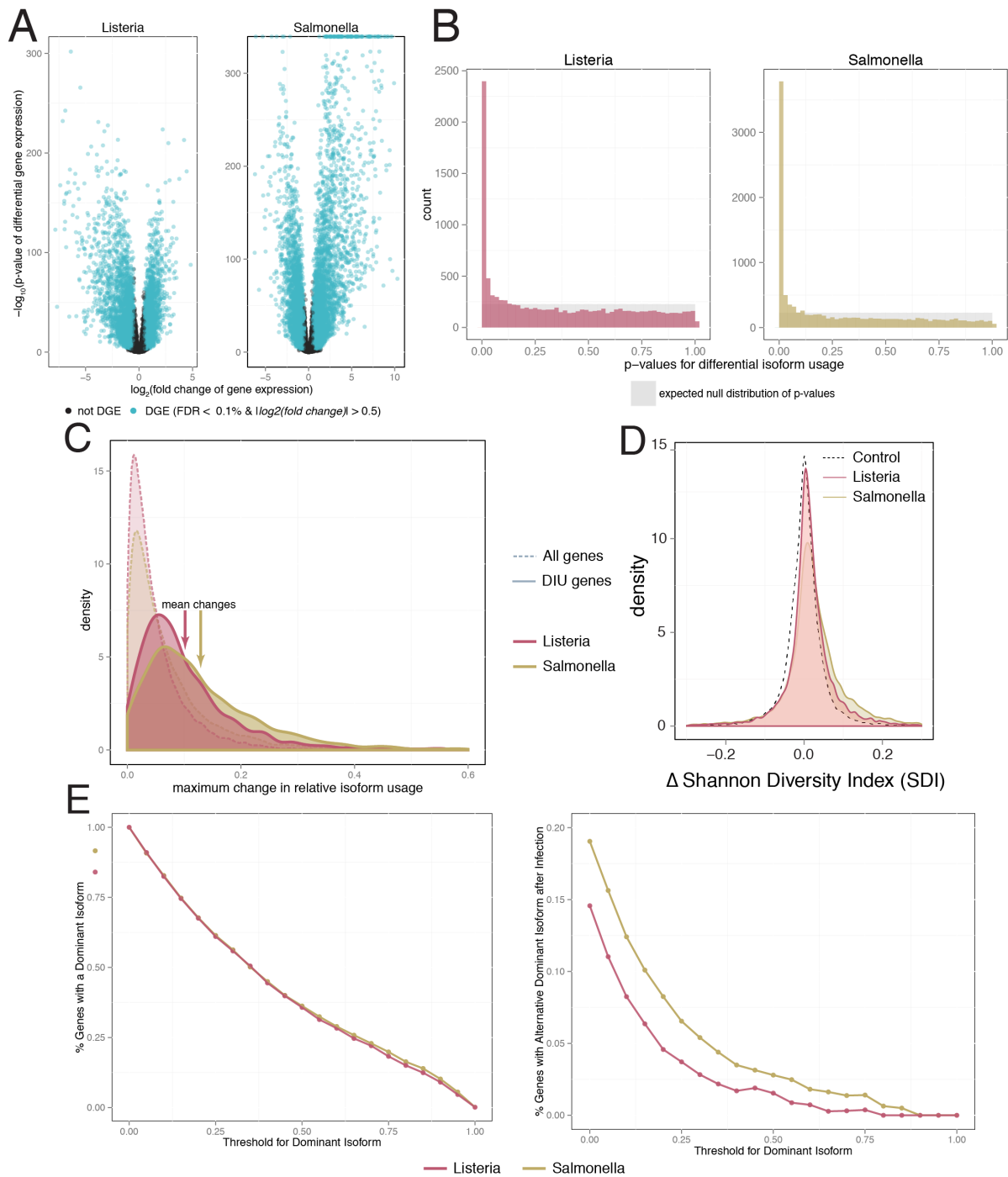

**S1 Fig. Differential isoform usage after infection**

Supplement: S1 Fig — (A) Volcano plots of differential expressionafter infection with Listeria and Salmonella in the left and right panels, respectively.–log10 p-values (y-axis) testing for differential expression are plotted against average log2 fold changes in expression levels (x-axis) for genes that are not differentially expressed (black) and genes that with significant differential expression after infection (FDR ≤ 0.1% and |log2(fold change)| ≥ 0.5; blue). (B) Distribution of p-values for the differential isoform usage test upon infection with Listeria and Salmonella. Expected distribution of p-values under the null hypothesis of no significant difference between the mean relative isoform usage is shown in grey. (C) A comparison of DIU effect sizes between DIU genes at 1% FDR (following infection with Listeria in dark pink and Salmonella in gold) and the background of all genes (lighter colors). Effect sizes are defined as maximum change in relative isoform abundances per gene upon infection. (D) Distributions of ΔShannon diversity index (ΔSDI) after infection with Listeria and Salmonella. Null distribution (black dotted line) was generated by permuting samples across conditions. (E) The percentage of genes with a dominant isoform before infection (left) and the fraction of these genes where the dominant isoform changes after infection (right). (PDF) [file pgen.1006338.s002.pdf]

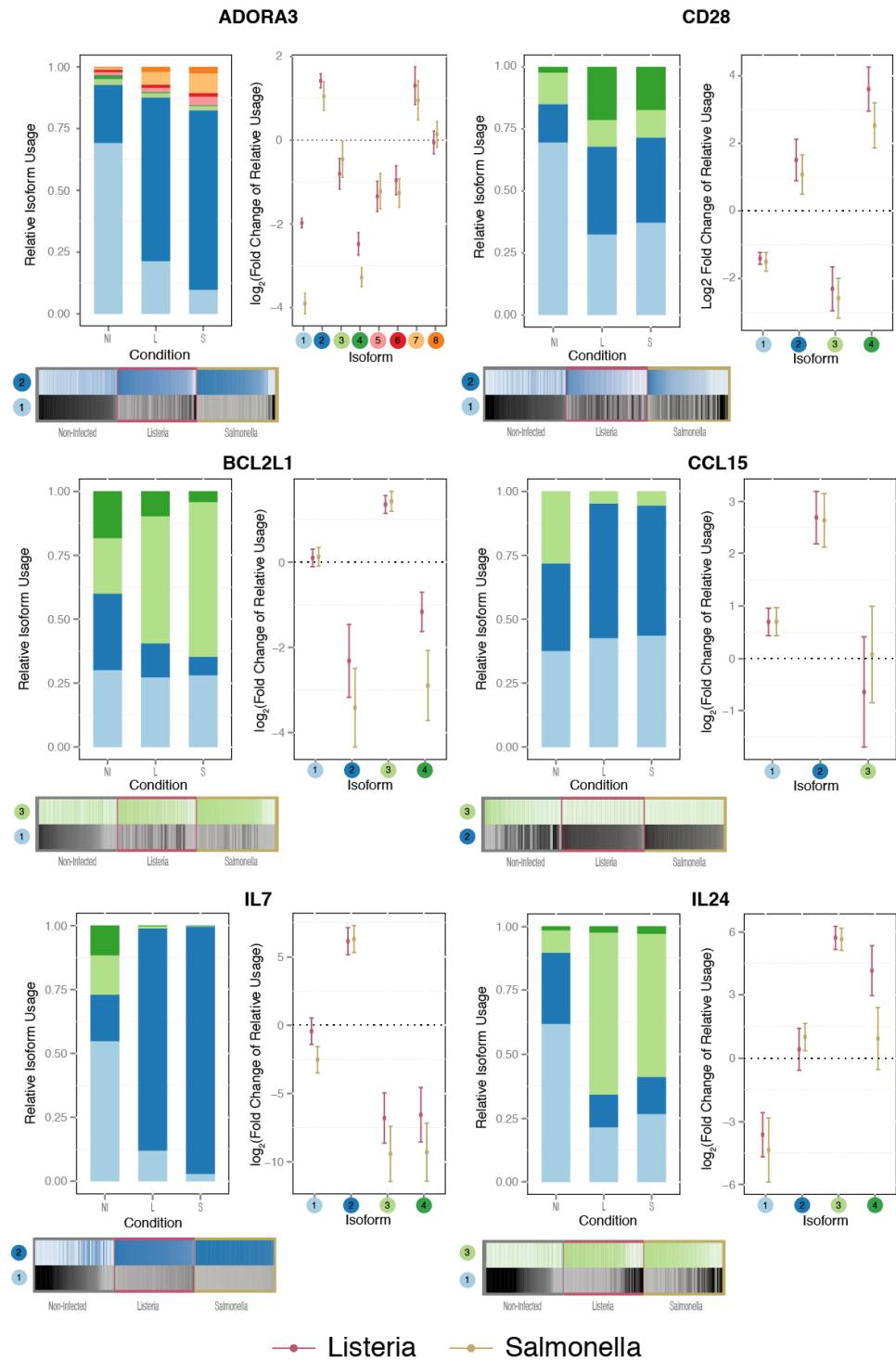

**S2 Fig. Representative examples of immune-related genes with significant DIU after infection.**

Supplement: S2 Fig — Heatmaps below each example represent the variation in isoform usage across the 60 individuals, where each vertical bar represents one individual. The dominant isoform in non-infected samples is represented in grey, while the predominant isoform in infected samples is in a color. Darker bars represent increased relative usage, while lighter bars represent decreased relative usage. (PDF) [file pgen.1006338.s003.pdf]

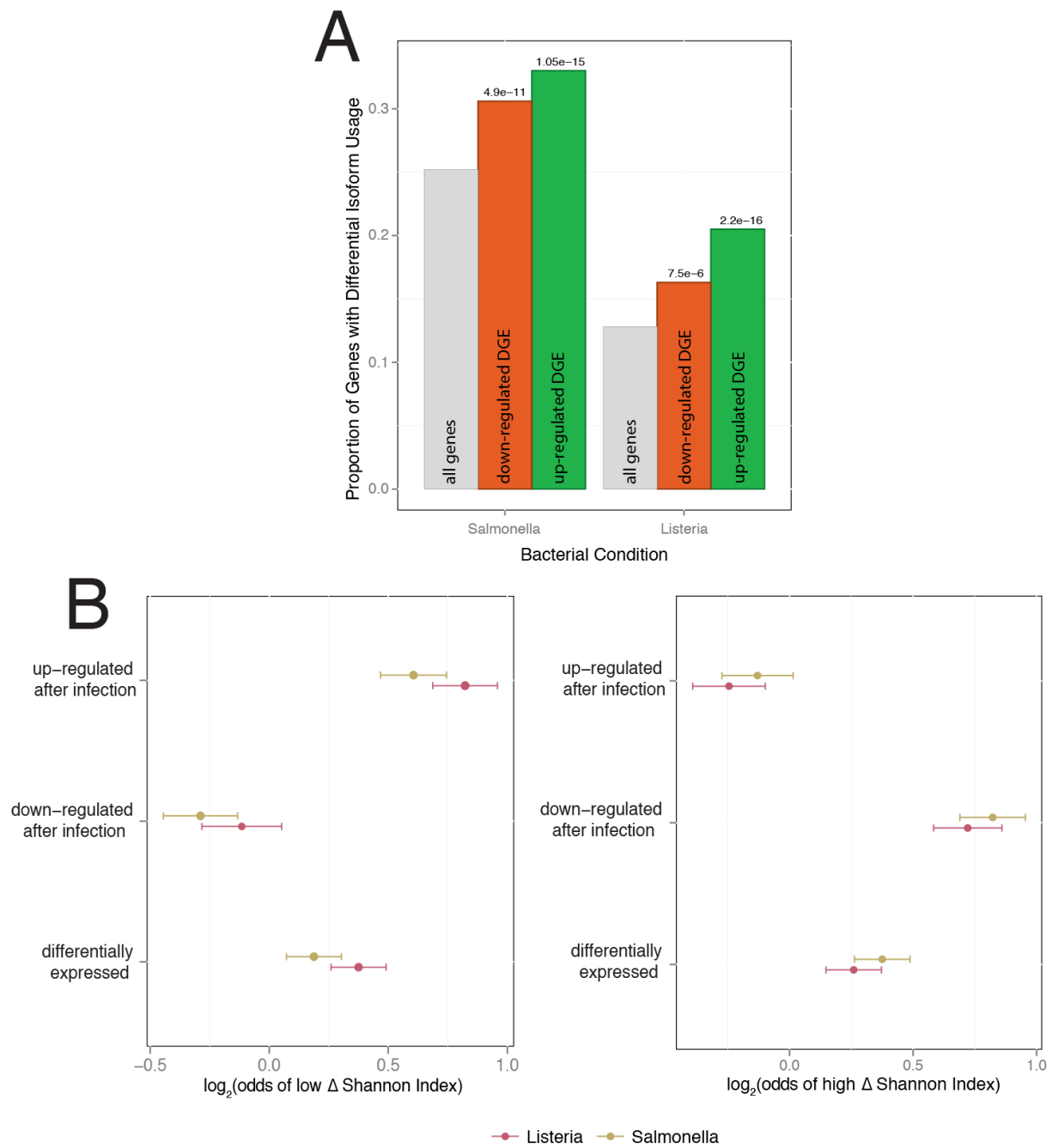

**S3 Fig. Gene expression and differential isoform diversity after infection.**

Supplement: S3 Fig — (A) Proportion of genes with differential isoform usage (y-axis) among all genes (grey), differentially expressed genes that are down-regulated after infection (orange), and differentially expressed genes that are up-regulated after infection (green). (B) log2 fold changes (with standard error bars) for odds of low ΔShannon and high ΔShannon among the set of all the differentially expressed genes, up-regulated differentially expressed genes, and down-regulated differentially expressed genes, following infection with Listeria (dark pink) and Salmonella (gold). (PDF) [file pgen.1006338.s004.pdf]

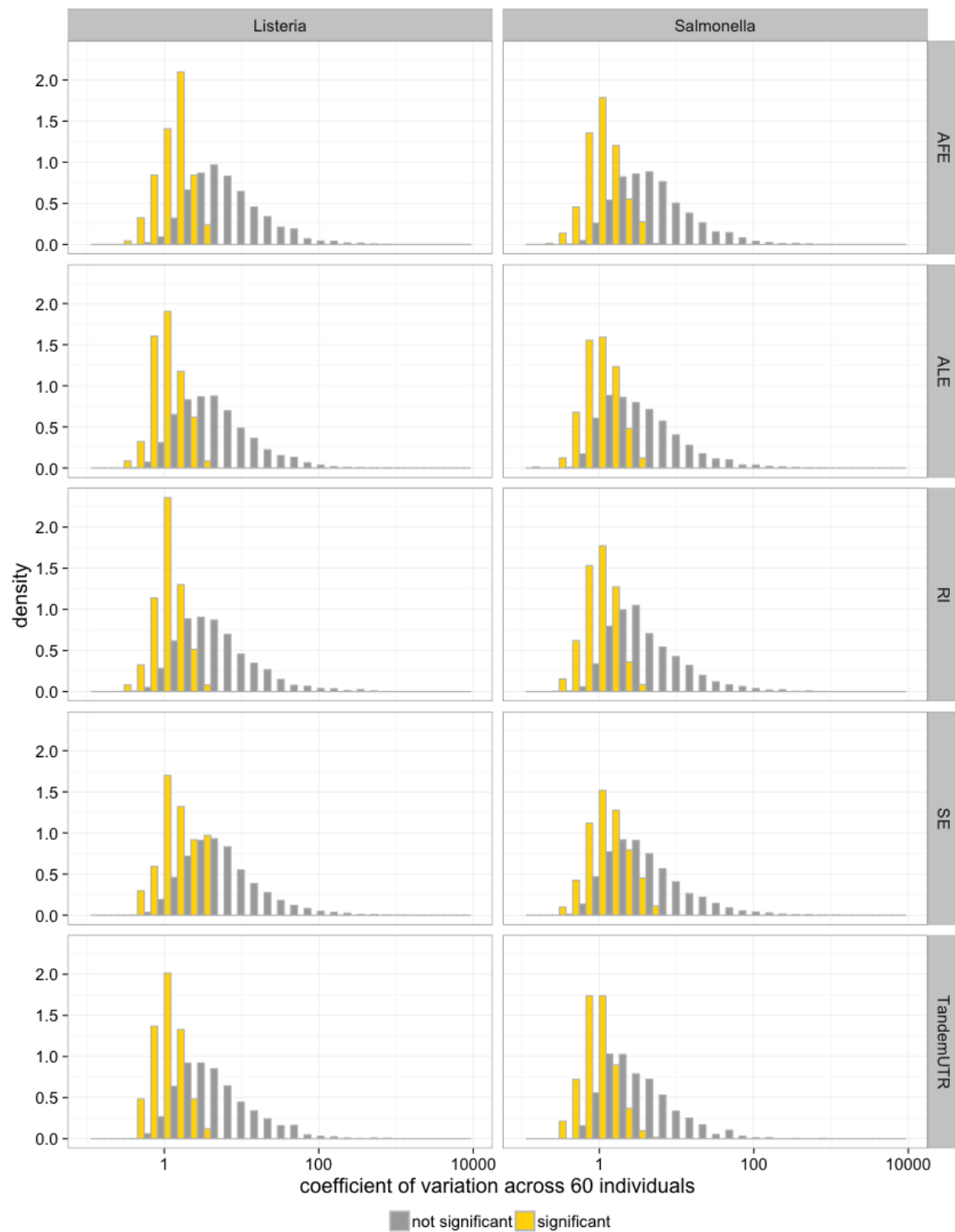

**S4 Fig. Variance in RNA processing across 60 individuals.**

Supplement: S4 Fig — Distribution of coefficient of variation (x-axis) in ΔΨ values for each isoform in a given RNA processing category. (PDF) [file pgen.1006338.s005.pdf]

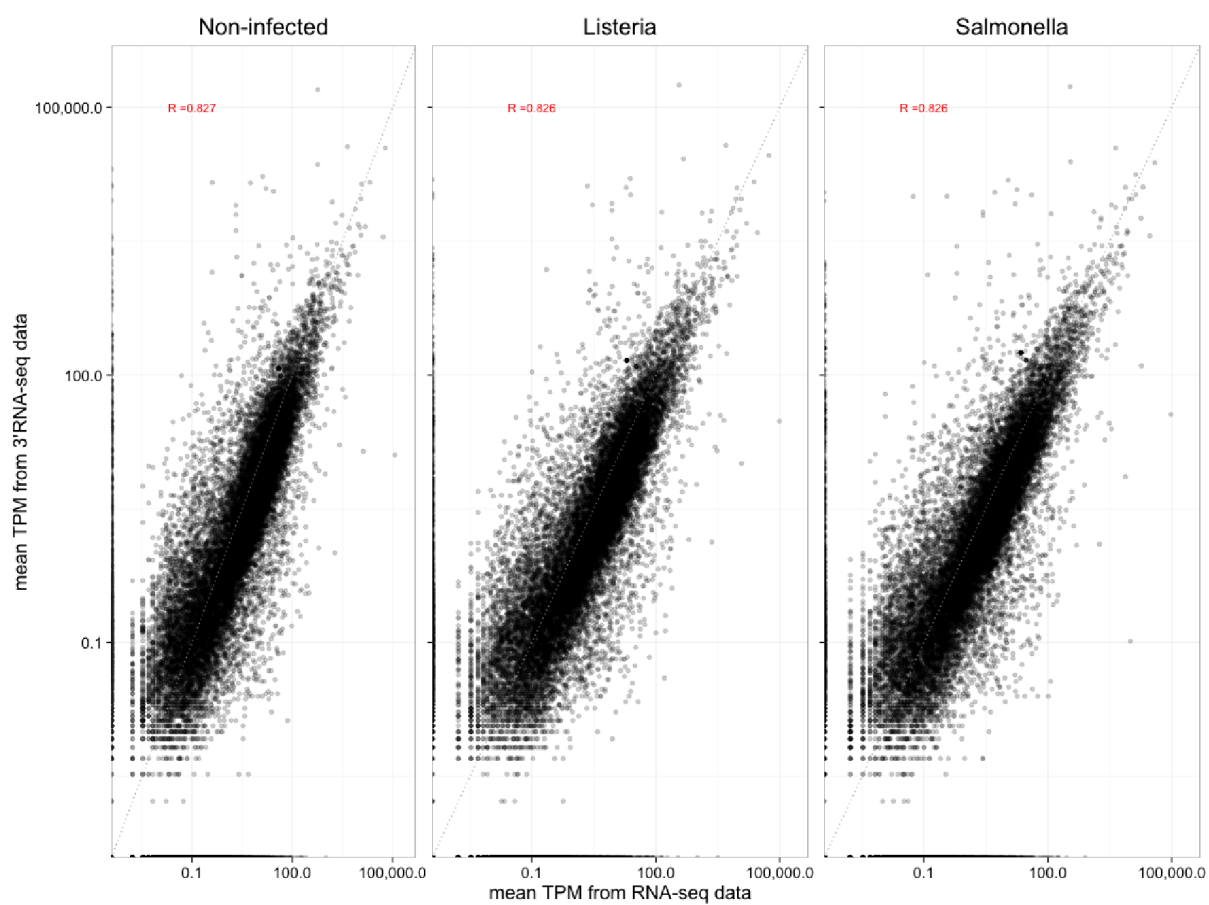

**S6 Fig. Relationship between 3'RNA-seq and RNA-seq data.**

Supplement: S6 Fig — Correlations between TPMs from 3' RNA-seq data (y-axis) and TPMs from RNA-seq data (x-axis). (PDF) [file pgen.1006338.s007.pdf]

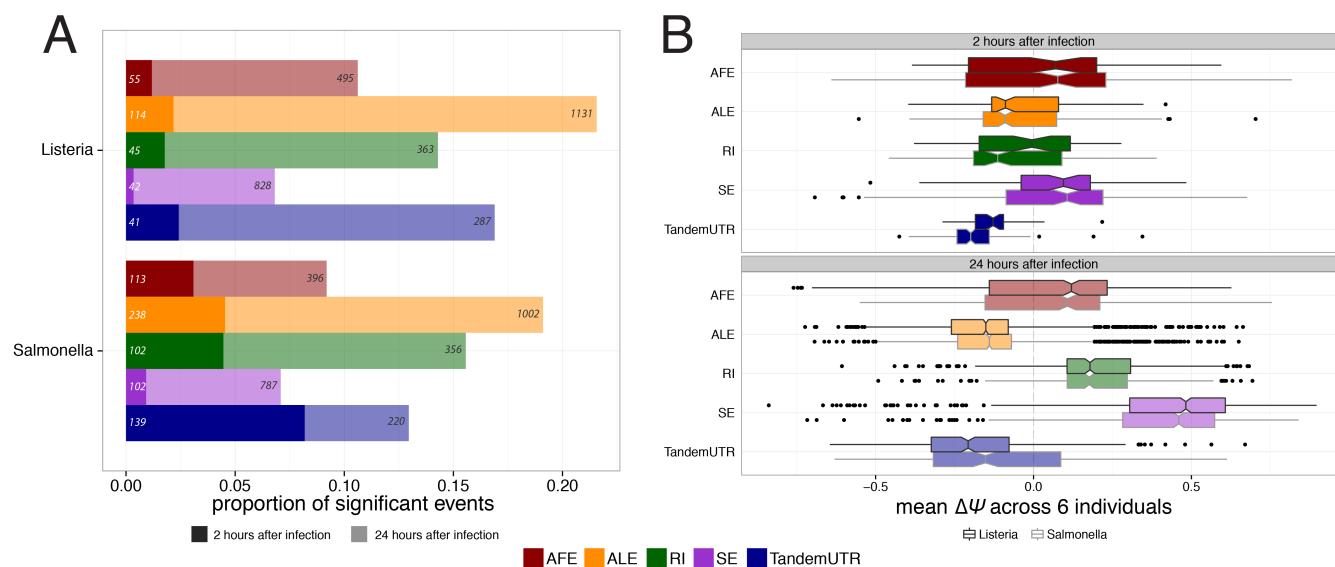

**S7 Fig. RNA processing changes 24hr after infection.**

Supplement: S7 Fig — (A) Proportion of significantly changing events (x-axis) after 2 hours of infection (dark colors) and 24 hours of infection (light colors) with either Listeria (top) or Salmonella (bottom). Numbers indicate the significant events at corresponding timepoints using only the 6 individuals used for these cross-timepoint analyses. (B) Distribution of ΔΨ values (x-axis) for significantly changing events in each event type after either 2 hours (top) or 24 hours (bottom) of infection. (PDF) [file pgen.1006338.s008.pdf]

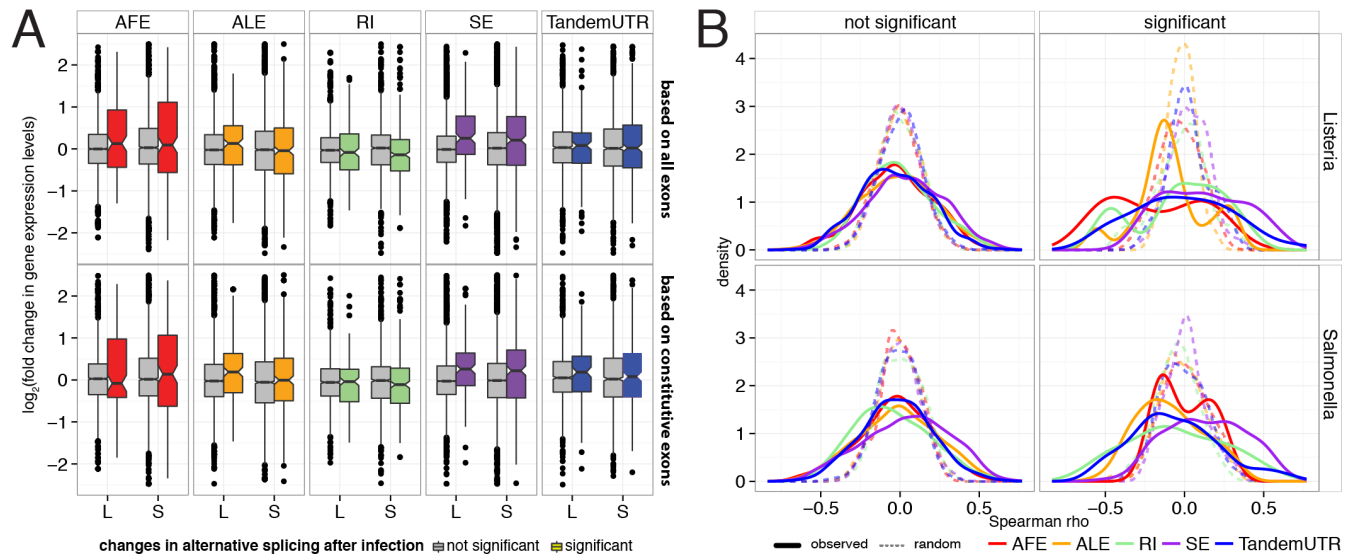

**S8 Fig. Relationships between RNA processing and gene expression changes after infection.**

Supplement: S8 Fig — (A) Distributions of overall fold changes in gene expression (y-axis, log2 scale) for genes that have significant splicing changes in each event type (colored boxplots) and genes with no splicing changes after infection (grey). Gene expression values are calculated using either full transcript models (top) or only constitutively included exons (bottom). (B) Distribution of spearman correlations between the ΔΨ of an event and the fold change in gene expression, across individuals, for events that are not significantly changing after infection (left) and significantly changing events (right). Solid lines represent the observed values and dotted lines represent a distribution of correlations after permuting the correspondence between ΔΨs and fold changes in gene expression. (PDF) [file pgen.1006338.s009.pdf]

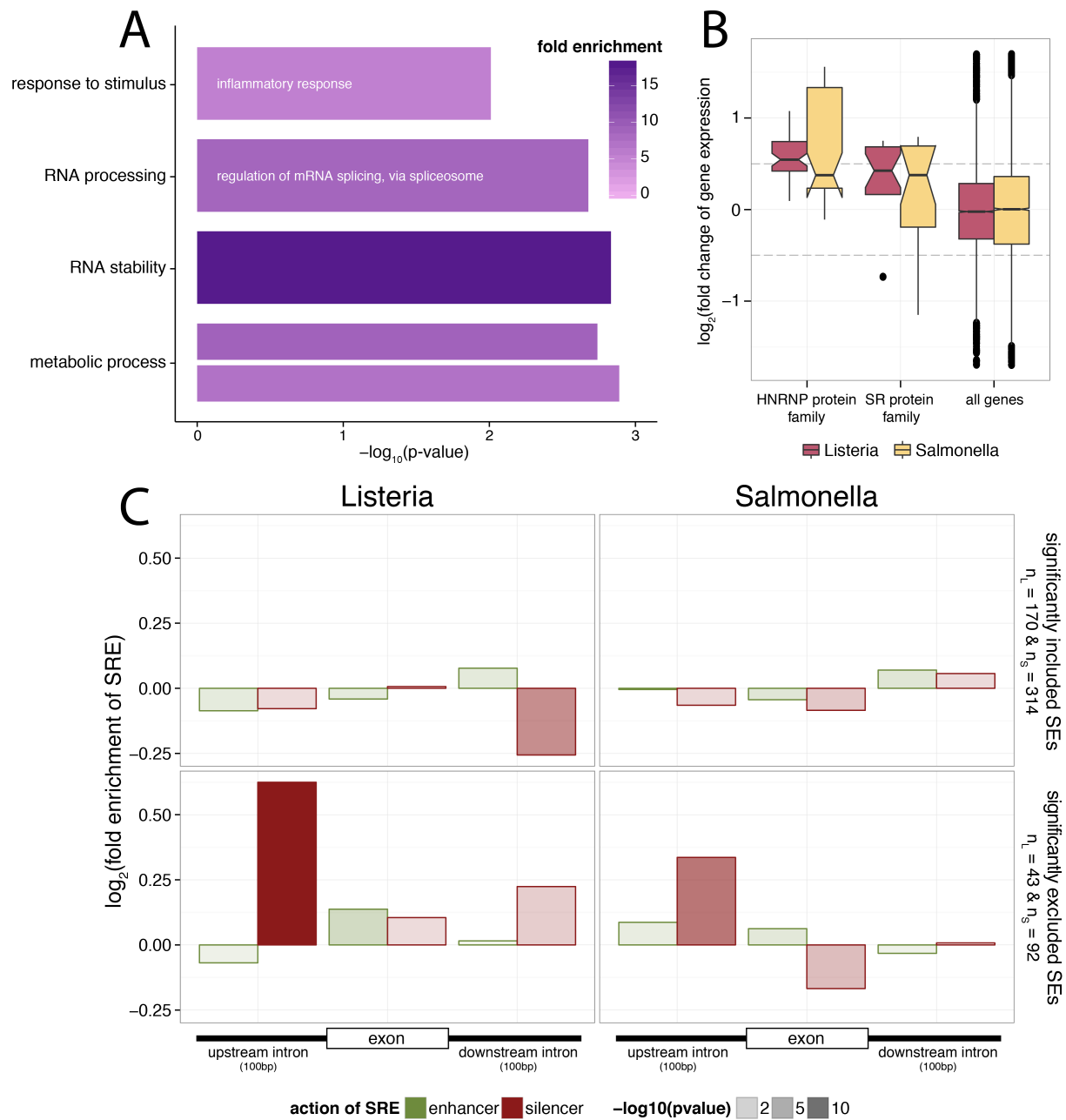

**S9 Fig. Characteristics of significantly changing skipped exon events.**

Supplement: S9 Fig — (A) Significantly enriched gene ontology categories for genes with significant skipped exon changes after infection. Color indicates the fold enrichment of the number of observed genes relative to the number of genes expected to be in that category. (B) Fold changes in gene expression (y-axis, log2 scale) after infection for 2 families of splicing factors (hnRNPs and SR proteins) relative to a background distribution of fold changes in all genes (C) Fold enrichments of splicing regulatory elements (SREs) in exonic regions and surrounding intronic regions (±100bp). SREs assessed included exonic splicing enhancers (green in exons), exonic splicing silencer (red in exons), intronic splicing enhancers (green in introns), and intronic splicing silencers (green in introns). Enrichments were calculated separately for significantly included skipped exons (top) and significantly excluded skipped exons (bottom). Shading of the bars indicates the significance of the enrichment. (PDF) [file pgen.1006338.s010.pdf]

**A**

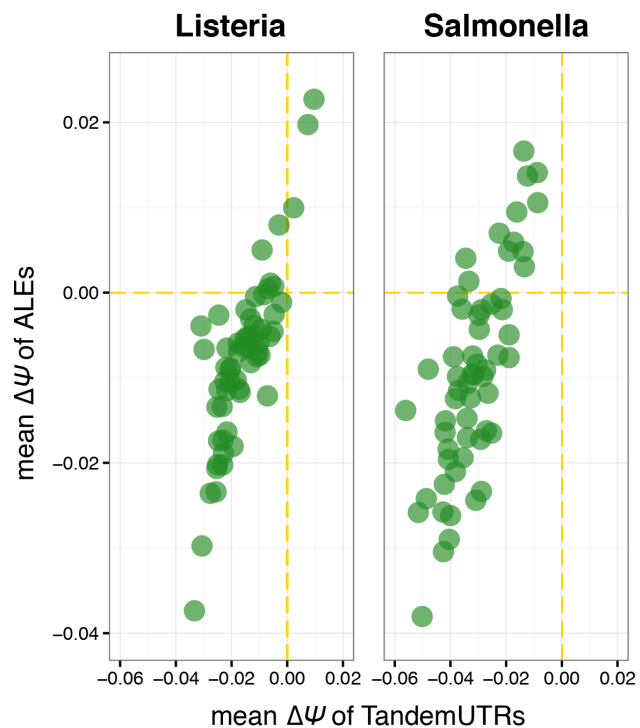

**B**

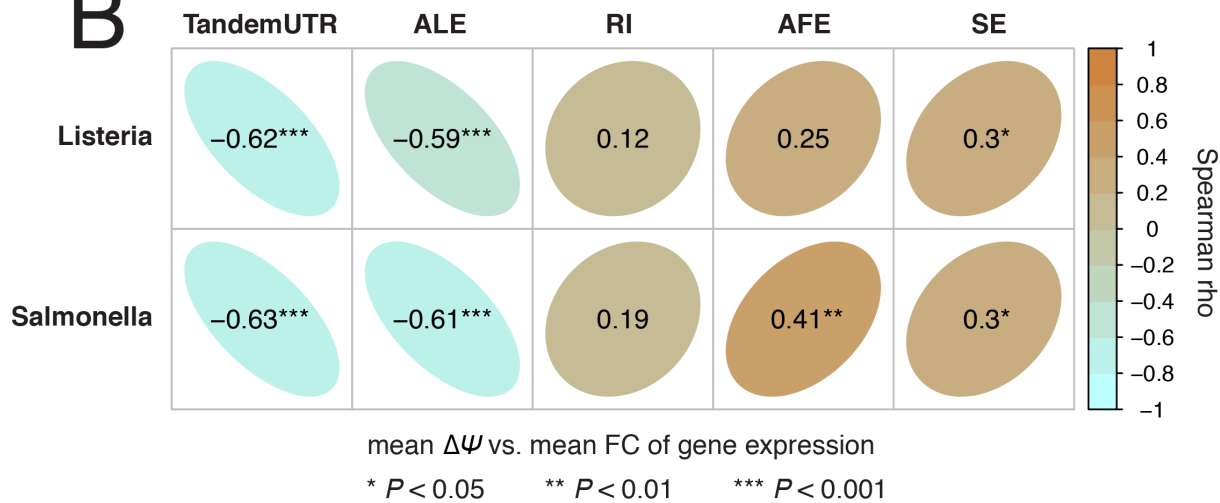

**S10 Fig. Inter-individual variation of global shifts in alternative splicing.**

Supplement: S10 Fig — (A) Correlations between the mean ΔΨ values per individual for ALEs (x-axis) and TandemUTRs (y-axis). (B) Spearman correlations between the mean ΔΨ value per individual and the mean fold change of gene expression per individual for corresponding genes. (PDF) [file pgen.1006338.s011.pdf]

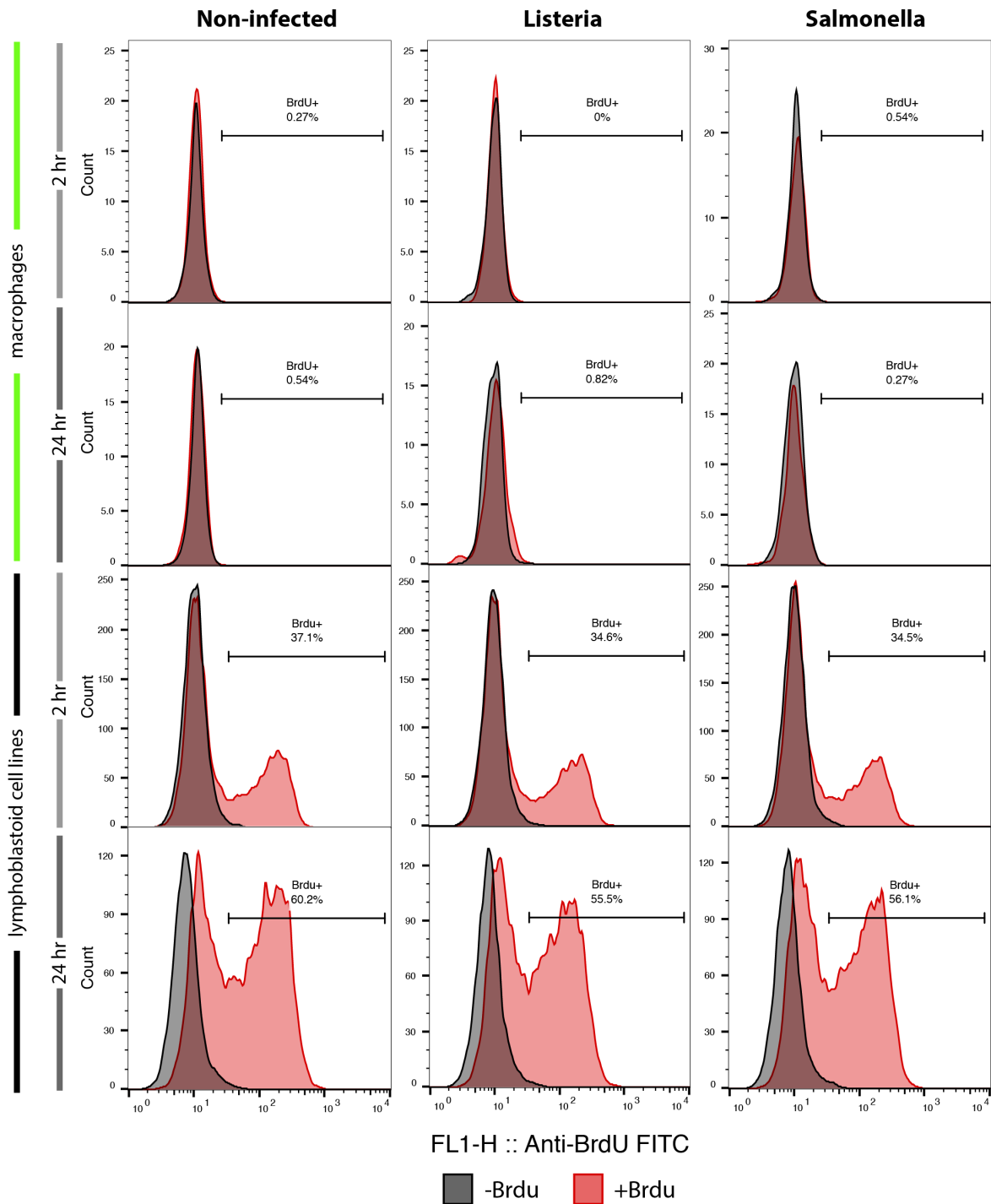

**S12 Fig. Cellular proliferation after bacterial infection.**

Supplement: S12 Fig — BrdU cell proliferation assay in macrophages (top panel) and LCLs (bottom panel) in non-infected cells and in cells infected with Listeria or Salmonella for both 2 and 24 hours. BrdU incorporates into newly synthesized DNA and therefore the quantity of BrdU incorporated into cells (x-axis) is a direct indication of cell proliferation. No evidence for cellular proliferation was observed in macrophages, in contrast to the high rates of proliferating cells observed in our positive control population of LCLs. (PDF) [file pgen.1006338.s013.pdf]

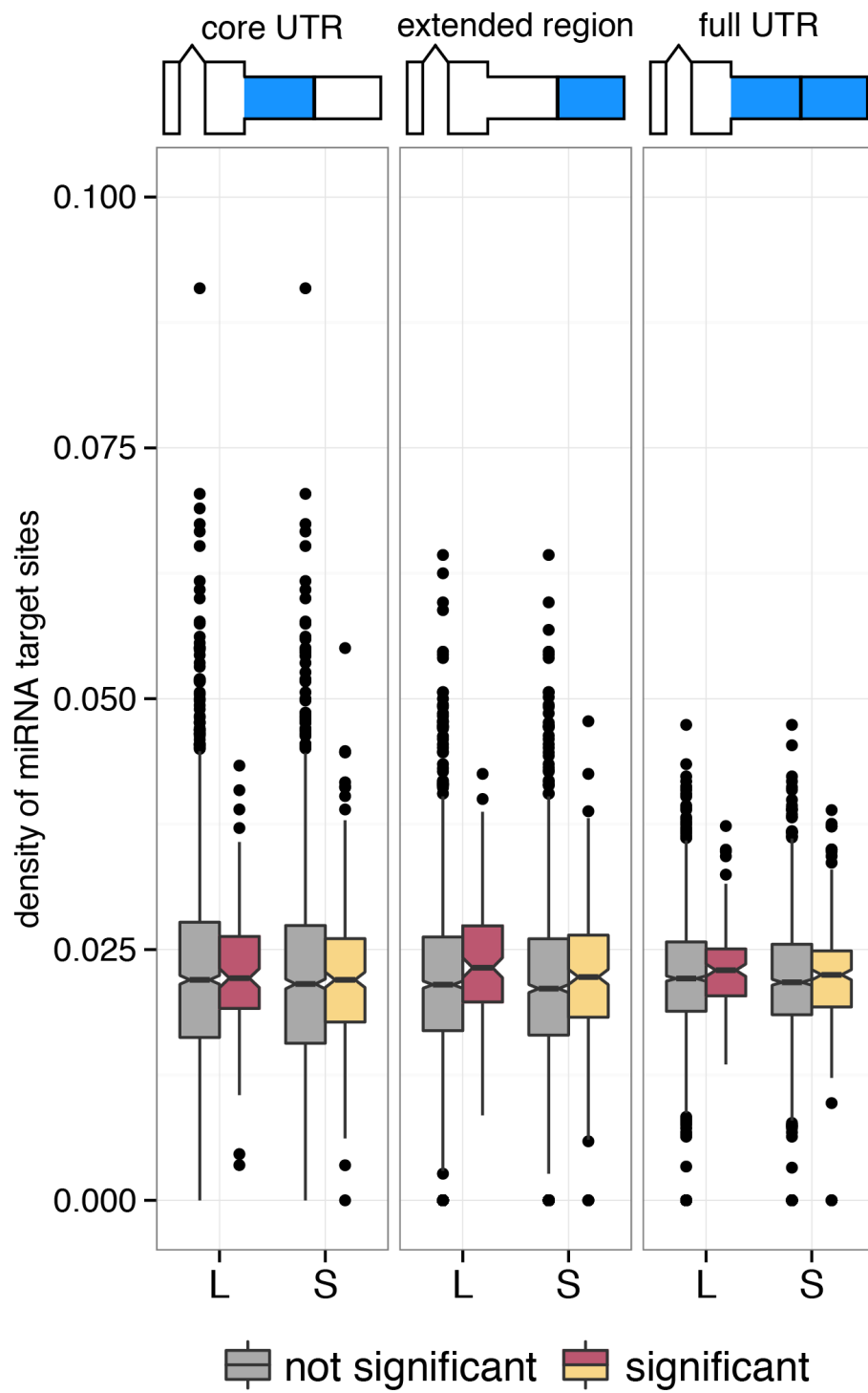

**S13 Fig. Density of miRNA target sites in 3' UTR regions.**

Supplement: S13 Fig — Barplots in grey indicate Tandem 3’ UTRs that are not changing after infection, while colored barplots indicate Tandem 3’ UTRs that are significantly changing after either Listeria (pink) or Salmonella (yellow) infections. (PDF) [file pgen.1006338.s014.pdf]

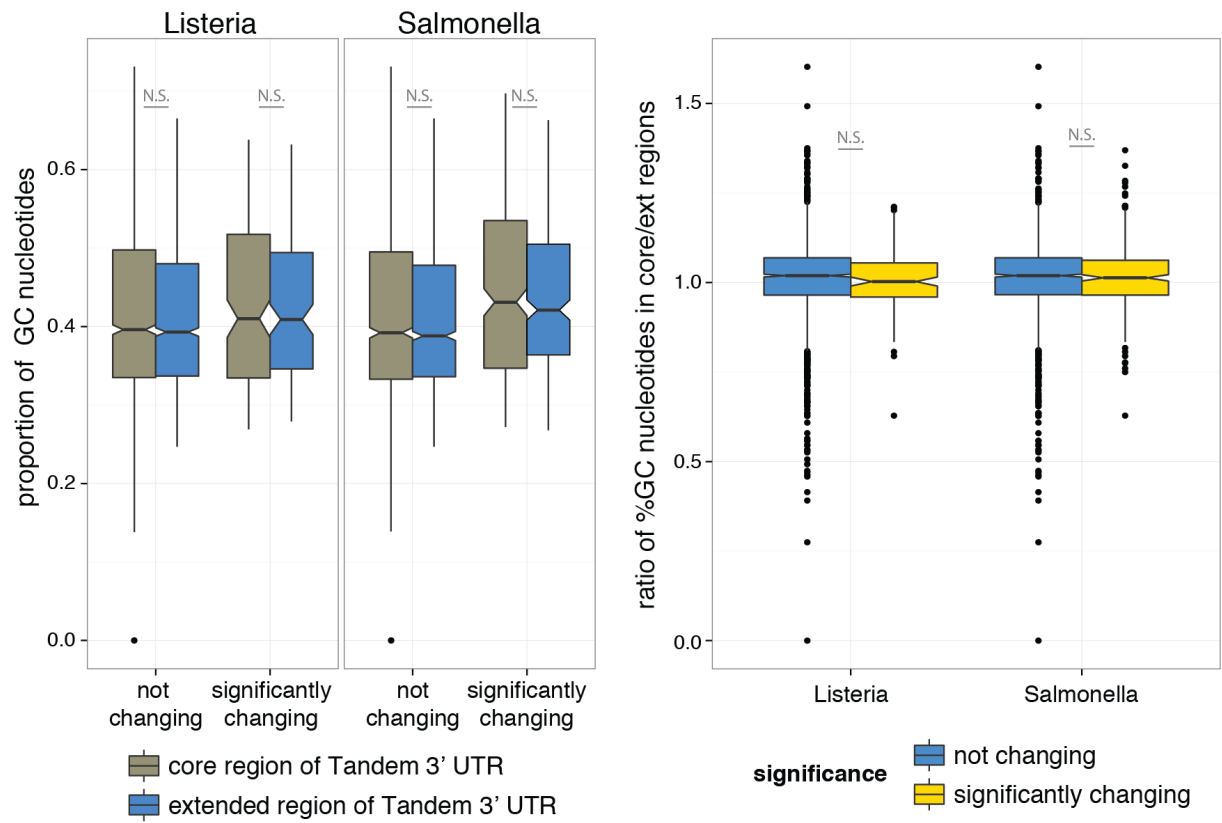

**S14 Fig. Nucleotide composition of Tandem 3' UTR regions.**

Supplement: S14 Fig — The distribution of GC content in core (brown) and extended (blue) regions of Tandem 3' UTRs that are either not changing or significantly changing after infection (left panel). While there 3' UTRs that are significantly changing generally have greater overall GC content, this is true for both the core and extended regions of the 3’ UTRs. Thus, the distributions of relative GC content when comparing the regions is distributed around 1 for both significantly changing (yellow) and not changing (blue) Tandem 3' UTRs (right panel). (PDF) [file pgen.1006338.s015.pdf]

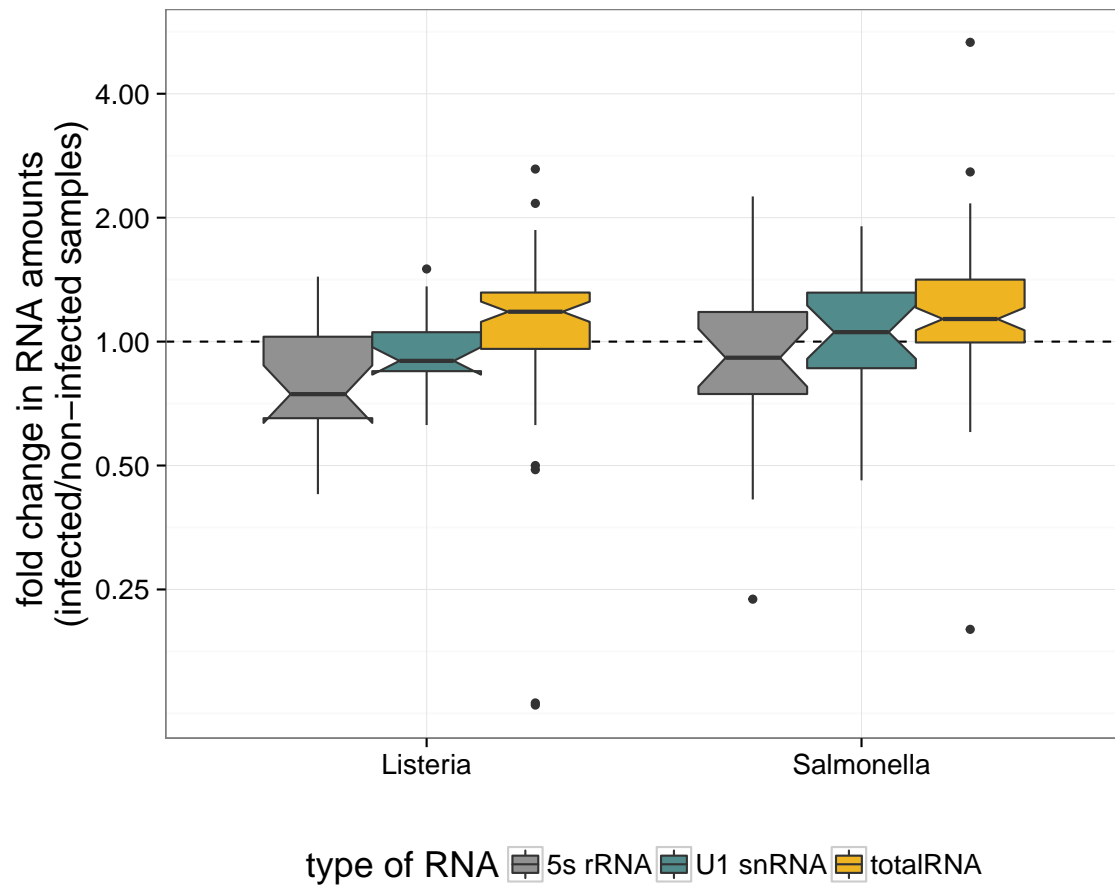

**S15 Fig. Investigating effects of telescripting after infection.**

Supplement: S15 Fig — Distributions of fold changes in 5s rRNA (grey, n = 20), U1 (green, n = 20), and total RNA (yellow, n = 60) concentrations after infection. 5s rRNA concentrations were calculated by taking the ΔCT value from qPCR measurements across 20 samples. Relative U1 snRNA concentrations were calculated by taking the ΔΔCT values from qPCR measurements across 20 samples, where U1 snRNA is measured relative to 5s rRNA concentrations in the same samples. Total RNA concentrations were estimated from Nanodrop measurements of RNA extraction yields across all 60 samples in our study. All samples (both non-infected and infected) were plated at exactly the same macrophage cellular density at the start of the experiment and we have confirmed that these macrophages do not proliferate either before or after infection (S12 Fig). There are no significant shifts in the distribution of 5s rRNA or U1 snRNA fold changes after infection (t-test for null of μ = 1, both P > 0.01 for 5s rRNA and both P > 0.2 for U1 snRNA), while the distribution of total RNA fold changes are significantly increased after infection (t-test for null of μ = 1, P < 0.001 for both Listeria and Salmonella samples). (PDF) [file pgen.1006338.s016.pdf]
