# Supplementary material for: Widespread Shortening of 3’ Untranslated Regions and Increased Exon Inclusion Are Evolutionarily Conserved Features of Innate Immune Responses to Infection
Source: PLoS Genet. 2016 Sep 30;12(9):e1006338. doi: 10.1371/journal.pgen.1006338 (PMC5045211; doi:10.1371/journal.pgen.1006338)
Supplement: S5 Fig — Sashimi plots for two genes, PTK2B and STK40, that have significant changes in skipped exon usage both after 2 hours and 24 hours of infection with either bacteria. (PDF) [file pgen.1006338.s006.pdf]

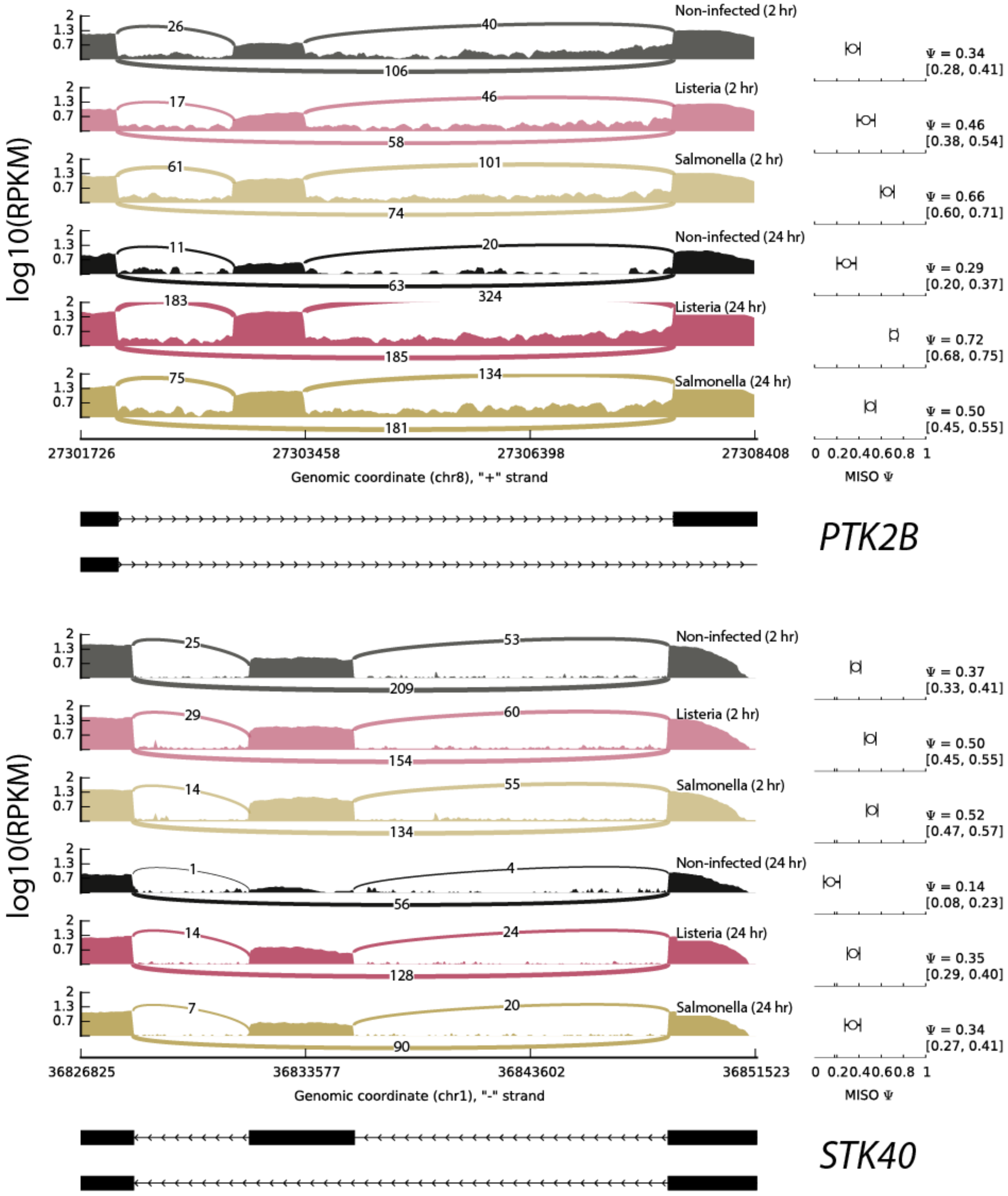

S5 Fig. Two representative examples of genes with significant skipped exon changes after infection.
