# Supplementary material for: Widespread Shortening of 3’ Untranslated Regions and Increased Exon Inclusion Are Evolutionarily Conserved Features of Innate Immune Responses to Infection
Source: PLoS Genet. 2016 Sep 30;12(9):e1006338. doi: 10.1371/journal.pgen.1006338 (PMC5045211; doi:10.1371/journal.pgen.1006338)
Supplement: S11 Fig — For both Tandem UTRs (A) and Skipped exons (B), the top panel is a scatter plot of Spearman correlations between the individual-specific mean ΔΨ values and individual-specific fold change of gene expression values for all expressed genes (grey) in both Listeria (x-axis) and Salmonella (y-axis). Genes with significant correlations (FDR ≤ 1%) in both Listeria and Salmonella conditions are plotted in black, and those factors with known RNA-binding properties are colored by their functional category. The bottom panels show distributions of the average Spearman correlation values for each of the RNA-binding functional categories with significant correlations. (PDF) [file pgen.1006338.s012.pdf]

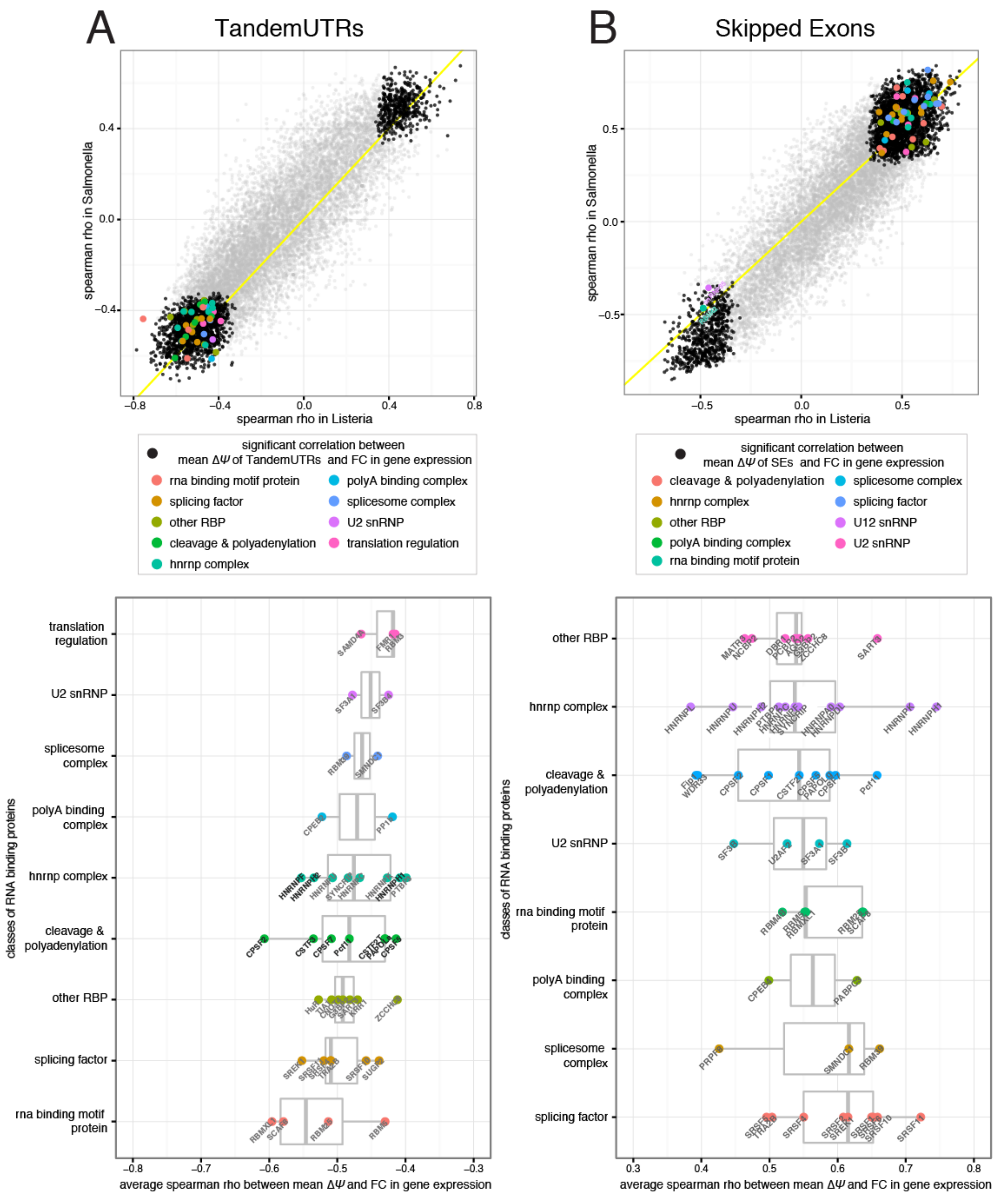

S11 Fig. Identifying RNA binding factors that might be involved in *trans*-regulation of RNA processing after infection.
